# Water Uptake, Thin-Film Characterization, and Gravimetric pH-Sensing of Poly(vinylphosphonate)-Based Hydrogels

**DOI:** 10.1021/acsami.4c17704

**Published:** 2024-12-20

**Authors:** Anton S. Maier, Matjaž Finšgar, Beatrice De Chiara, Rupert Kargl, Bernhard Wolfrum, Karin Stana Kleinschek, Bernhard Rieger

**Affiliations:** † Technical University of MunichTUM School of Natural Sciences, Department of Chemistry, WACKER-Chair of Macromolecular Chemistry, Lichtenbergstraße 485748 Garching, Germany; ‡ Faculty of Chemistry and Chemical Engineering, University of Maribor, 2000 Maribor, Slovenia; § Technical University of MunichTUM School of Computation, Information and Technology, Munich Institute of Biomedical Engineering, Department of Electrical Engineering, Neuroelectronics, Hans-Piloty-Str. 1, 85748 Garching, Germany; ∥ Graz University of TechnologyInstitute for Chemistry and Technology of Biobased Systems (IBioSys), Stremayrgasse 98010 Graz, Austria

**Keywords:** superabsorbent hydrogels, pH-responsiveness, hydrogel thin films, quartz crystal microbalance, pH sensor, reversible swelling, rare earth metal-mediated group-transfer polymerization

## Abstract

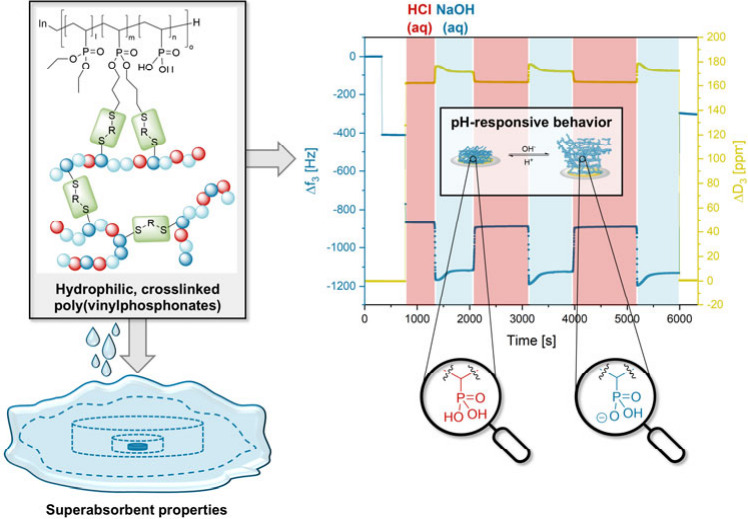

Herein, novel, superabsorbent, and pH-responsive hydrogels obtained by the photochemical cross-linking of hydrophilic poly(vinylphosphonates) are introduced. First, statistical copolymers of diethyl vinylphosphonate (DEVP) and diallyl vinylphosphonate (DAlVP) are synthesized via rare earth metal-mediated group-transfer polymerization (REM-GTP) yielding similar molecular weights (*M*_n,NMR_ = 127–142 kg/mol) and narrow polydispersities (*Đ* < 1.12). Subsequently, polymer analogous transformations of P(DEVP-*stat*-DAlVP) introduced vinylphosphonic acid (VPA) units into the polymers. In this context, the partial dealkylation of the polymers revealed a preference for DAlVP hydrolysis, which was observed via ^1^H NMR spectroscopy and explained mechanistically. Furthermore, the P(DEVP-*stat*-DAlVP-*stat*-VPA) polymers were cross-linked under photochemical reaction conditions (λ = 365 nm) via thiol–ene click chemistry, yielding superabsorbent hydrogels with water uptakes up to 150 ± 27 g (H_2_O)/g (hydrogel). Regarding water absorption, evident structure–property relationships between cross-linking density, polarity, and swelling behavior were found. Finally, the pH-responsiveness of thin films of these hydrogels was investigated. In this regard, films with a thickness of 39.4 ± 2.33 nm determined via profilometry were spin-coated on sensors of a quartz crystal microbalance with dissipation monitoring (QCM-D) and thoroughly characterized by atomic force microscopy (AFM). QCM-D measurements exposing the hydrogel films to different aqueous media revealed different swelling states of the hydrogels depending on the pH values (1, 6, 10, and 13) of the surrounding environment, as reflected by corresponding frequency and dissipation values. The hydrogels exhibited fully reversible swelling and deswelling upon switching between pH 1 and 13 (three cycles), sustaining the harsh conditions without erosion from the gold surface and thus acting as a gravimetric sensor discriminating between the two pH values. The high stability of the films on the gold surfaces of QCM-D sensors was explained by anchoring of the P(DEVP-*stat*-DAlVP-*stat*-VPA) networks through the dithiol cross-linker as confirmed by detailed X-ray photoelectron spectroscopy (XPS) and time-of-flight secondary ion mass spectrometry (ToF-SIMS) studies.

## Introduction

In recent years, stimuli-responsive hydrogels have emerged as a promising area of research in biomedical fields such as controlled drug delivery and tissue engineering due to their high biocompatibility and resemblance to biological tissues.^1−3^ In this context, employing these materials enables the release of an active pharmaceutical ingredient, such as a protein or a drug, in a controlled manner through an external trigger or changes in the surrounding conditions.^4,5^ Hydrogels in stimuli-responsive applications often serve as scaffolds reacting to environmental changes, e.g., differences in the pH value,^6−8^ temperature,^9,10^ light,^11,12^ electricity,^13,14^ and the presence of biomolecules.^15^ Beyond biomedicine, several other areas employ cross-linked materials with these properties. For instance, in the field of actuators and soft robotics, these components can undergo mechanical motion upon external stimulation, or in the fabrication of sensors, hydrogels with the ability to respond to variations in an ambient medium are up-and-coming candidates.^16−20^ The swelling behavior and other material characteristics of synthetic and natural hydrogels are often influenced by the pH value of the surrounding medium, affecting the charge of acidic or basic groups in the polymer networks. Notable examples of pH-responsive moieties in hydrogels include acrylic acid-containing networks, along with chitosan and hyaluronic acid derivatives as representatives of biopolymers.^19,21−23^ The potential of certain materials reacting to changes in the pH value is highlighted by their widespread implementation in numerous areas of research and everyday life. These include smart and antibacterial coatings,^22,24−27^ smart packaging,^28^ actuators and sensors,^16,17,19,20^ and lab-on-a-chip applications,^29^ among others, therefore creating incentives for further research toward the discovery of novel materials with these properties. Before application, the pH-dependent behavior of newly established hydrogels is typically investigated by analyzing the water uptake of different samples across a range of pH values. Additional insights into the response of thin films of cross-linked, pH-sensitive networks can be obtained through a quartz crystal microbalance with dissipation monitoring (QCM-D), providing an online measurement of the swelling state as well as the viscoelastic properties of the sample.^23,30−32^ The water uptake of a hydrogel is usually governed by several factors, including the hydrophilicity of the polymers, the cross-linking density of the network, chain mobility, and external factors like the ionic strength of the medium used for swelling experiments.^33−35^ Further, the water uptake may be increased by introducing sodium salts of deprotonated acids through increased hydrophilicity and solvation of both anions and cations.^36^ In a recent study, we introduced hydrogels obtained through photochemical cross-linking of statistical copolymers consisting of diethyl vinylphosphonate (DEVP) and diallyl vinylphosphonate (DAlVP) applying thiol–ene click chemistry with dithiols as cross-linkers. The final materials exhibited widely tunable properties in terms of water uptake and mechanical strength, as well as good biocompatibility.^37^ The corresponding copolymers were obtained with excellent control over the polymer microstructure while maintaining narrow polydispersities by applying yttrium-catalyzed rare earth metal-mediated group-transfer polymerization (REM-GTP).^38^ This highly precise polymerization technique gives access to well-defined polymeric structures in terms of molecular weight and polydispersity through repeated 1,4-conjugate addition of Michael-type monomers.^39,40^ Further, REM-GTP enables a straightforward introduction of biologically active motifs and other substrates into allyl group-containing poly(vinylphosphonates) under mild conditions through postpolymerization thiol–ene click chemistry.^41,42^ As demonstrated in the study mentioned above, the functionalization of polymers with sodium salts of organic acids prior to photochemical cross-linking toward hydrogels was successfully applied, yielding materials with significantly increased swelling ratios.^36,37^ Another approach for increasing the water uptake worth exploring includes polymer modification rather than functionalization. In this context, the well-established transformation of poly(vinylphosphonates) with trimethylsilyl bromide (TMSBr) results in partial hydrolysis of the polymer side chains toward poly(vinylphosphonic acid) (PVPA). When applied to allyl-group containing poly(vinylphosphonates), this should generate cross-linkable polymers with increased hydrophilicity, potentially inducing pH-responsiveness into the final materials.^43^ In this study, different P(DEVP-*stat*-DAlVP) copolymers obtained via REM-GTP are subjected to polymer-analogous hydrolysis of the monomers toward statistical P(DEVP-*stat*-DAlVP-*stat*-VPA) terpolymers, which are cross-linked applying photoinitiated thiol–ene click reactions with 3,6-dioxa-1,8-octanedithiol. After being dried, the resulting hydrogels are thoroughly analyzed regarding water uptake. Further, in-depth characterization of thin films of these novel materials and detailed analyses of the pH-responsiveness through QCM-D measurements contribute to a profound understanding of the high application potential in actuators and sensors.

## Experimental Section

### Statistical Copolymerization of Diethyl Vinylphosphonate (DEVP) and Diallyl Vinylphosphonate (DAlVP)

The statistical copolymerization of DEVP and DAlVP was performed according to previously reported procedures.^37,41^ In a copolymerization experiment, the calculated amount of catalyst Cp_2_YCH_2_TMS(thf) (1.00 equiv) was dissolved in dry toluene. The calculated amount of initiator (1.10 equiv) was added to this solution, resulting in a yellow coloration. After 2 h, quantitative conversion of the catalyst toward the initiating species was confirmed via ^1^H NMR spectroscopy by withdrawing an aliquot of the reaction mixture (0.1 mL of solution +0.4 mL of C_6_D_6_), and a mixture of monomers in the desired ratio was added in one motion. The polymerization was stirred at room temperature for 2 h until a second aliquot (0.1 mL of solution + 0.4 mL of CD_3_OD) confirmed quantitative conversion via ^31^P NMR spectroscopy. Then, polymerization was stopped by adding 0.5 mL of undried MeOH, and the polymers precipitated from hexane. After centrifugation, the residues were taken up in 1,4-dioxane and subjected to lyophilization to yield the purified P(DEVP-*stat*-DAlVP) polymers as white solids.

### Partial Hydrolysis of P(DEVP-*stat*-DAlVP) toward Vinylphosphonic Acid (VPA)-Containing Terpolymers

The polymer-analogous hydrolysis of P(DEVP-*stat*-DAlVP) was performed according to a well-established procedure applying trimethylsilyl bromide (TMSBr).^43^ In an oven-dried Schlenk flask suitable for elevated pressures, P(DEVP-*stat*-DAlVP) was dissolved in dry CH_2_Cl_2_ (10 mL of solvent per 100 mg of polymer). Further, 0.30 equiv (or 0.15, as specified in the manuscript) of TMSBr was added, and the reaction mixture was refluxed for 16 h. Then, the solvent was removed in vacuo, and the residue was taken up in 20 mL of methanol, and 5 mL of 1 M hydrochloric acid (aq) was added. The resulting reaction mixture was stirred at room temperature for 2 h. Finally, the solvent was removed under reduced pressure, and the crude product was purified via dialysis against deionized water (8 kDa MWCO). Lyophilization yielded the partially hydrolyzed P(DEVP-*stat*-DAlVP-*stat*-VPA) polymers as white solids.

### Synthesis of Hydrogels from VPA-Containing Terpolymers

To obtain hydrogels from P(DEVP-*stat*-DAlVP-*stat*-VPA) terpolymers, the reaction conditions reported in an initial study were applied.^37^ In a typical experiment, 100 mg of polymer were dissolved in 0.3 mL of dioxane. For some polymers, small amounts of water were added to facilitate the solubilization of polymers. To this solution, the calculated amounts of cross-linker 3,6-dioxa-1,8-octanedithiol (2.50 equiv) and 0.40 equiv of the photoinitiator 2,2-dimethoxy-2-phenylacetophenone (DMPA) were added. After homogenization, the reaction mixture was cross-linked through UV irradiation (λ = 365 nm) for 60 min and dried to weight constancy in vacuo.

### Thin-Film Preparation

Stock solutions of P(DEVP-*stat*-DAlVP-*stat*-VPA) for spin-coating silicon wafers and QCM-D sensors were freshly prepared before each experiment. In this context, 25 mg of polymer was dissolved in 3 mL methanol and 0.5 mL distilled water, resulting in a homogeneous, clear solution. Subsequently, the calculated amounts of the cross-linker 3,6-dioxa-1,8-octanedithiol with respect to the allyl groups in the polymer (2.50 equiv per allyl group) and the photoinitiator 2,2-dimethoxy-2-phenylacetophenone (DMPA) (0.40 equiv per allyl group) were added to the solution. To prevent ambient light-induced cross-linking, all samples were prepared and stored in brown glass vials to protect them from surrounding light. The spin-coating of quartz crystals and silicon wafers was performed according to well-established procedures.^44,45^ The P(DEVP-*stat*-DAlVP-*stat*-VPA)-containing films were deposited on the static substrates by pipetting 50 μL of an 85 ppm (0.0085 wt %) polymer-containing stock solution in methanol/water (6/1) onto the surfaces of either the QCM-D sensors or the silicon wafers. Immediately after adding the stock solution, the substrates were rotated at a spinning speed of 4000 rpm with an acceleration of 2500 rpm/s for 60 s. In the final step, the films on the substrates were cross-linked by UV irradiation (λ = 365 nm) for 60 s.

### Workflow of QCM-D Measurements

In a typical QCM-D experiment, the frequencies and dissipation of cleaned and empty QCM-D sensors were monitored in ambient air for 5 min. Subsequently, the substrates were spin-coated with a freshly prepared polymer-, cross-linker-, and photoinitiator-containing solution, as described above.^44,45^ Next, the films on the substrates were cross-linked through UV irradiation (λ = 365 nm) for 1 min. The frequency change upon spin-coating was evaluated by measuring the sensors in air for another 5 min. Following that, the crystals were subjected to the different aqueous solutions described in the discussion, and the QCM-D response was monitored for the given timeframes, observing different swelling states of the hydrogel films. Finally, the QCM-D crystals were removed from the device, dried thoroughly with nitrogen gas to avoid mechanical removal of the films, and remeasured in air to confirm that no significant sample leaching occurred.

## Results and Discussion

### Polymer Synthesis and Modification

**Scheme 1:**
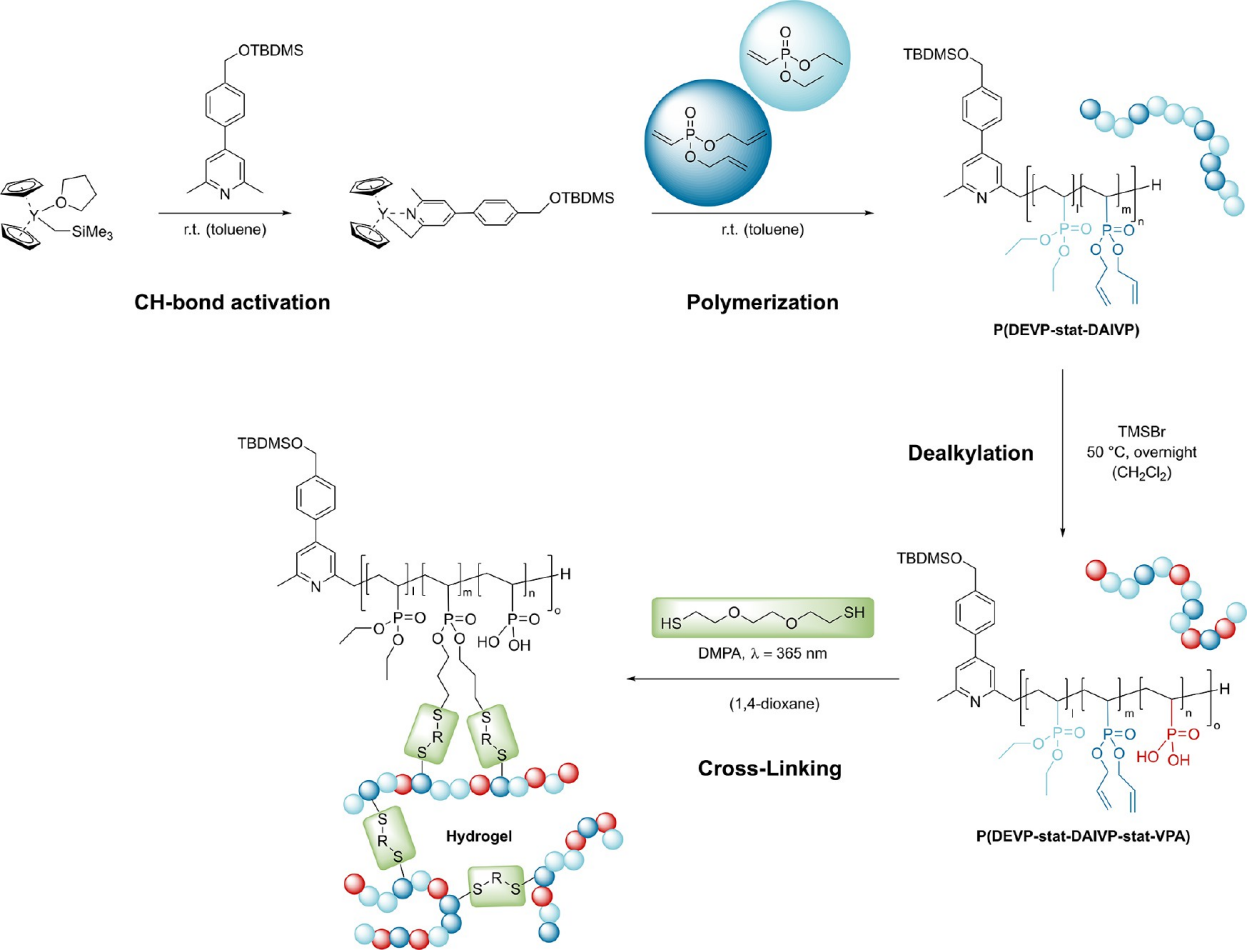
Overview of the Synthetic Pathway toward Highly Water-Absorbing Hydrogels CH-bond activation of a sym-collidine derivative with Cp_2_YCH_2_TMS(thf) towards the initiating complex (Step 1); polymerization through addition of the monomers diethyl vinylphosphonate (DEVP) and diallyl vinylphosphonate (DAlVP) (Step 2); polymer-analogous transformation of the copolymer side-chains towards vinylphosphonic acid (VPA) units (Step 3); crosslinking of P(DEVP-stat-DAlVP-stat-VPA) chains via photoinitiated thiol–ene click chemistry to highly hydrophilic hydrogels (Step 4).

As reported in previous studies, statistical copolymers from the water-soluble monomer diethyl vinylphosphonate (DEVP) and the cross-linkable monomer diallyl vinylphosphonate (DAlVP) were obtained through rare earth metal-mediated group-transfer polymerization (REM-GTP).^37^ After the quantitative conversion of the Cp_2_YCH_2_TMS(thf) catalyst toward the initiating species was achieved (Scheme 1, Step 1), as confirmed by ^1^H NMR spectroscopy in C_6_D_6_, a mixture of the monomers was added to initiate the polymerization (Scheme 1, Step 2). Following, once quantitative monomer conversion was confirmed via ^31^P NMR spectroscopy, the polymers were purified by precipitation and characterized by ^1^H NMR (Figure S1) and ^1^H DOSY NMR spectroscopy (Figure S3) as well as size-exclusion chromatography multiangle light scattering (SEC-MALS). An overview of the polymerization results of different P(DEVP-*stat*-DAlVP) copolymers is given in Table 1.

**Table 1:** Selected Polymerization Results of the Polymerization of Diethyl Vinylphosphonate (DEVP) and Diallyl Vinylphosphonate (DAlVP) Applying the Rare Earth Metal-Based Catalyst Cp_2_YCH_2_TMS(thf)^a^

Polymer	DAlVP content [%]^b^	DEVP content [%]^b^	*M*_n,NMR_ [kg/mol]^c^	IE^d^	Đ^e^
1	10.9	89.1	137	43	1.06
2	19.1	80.9	138	43	1.12
3	24.7	75.3	127	48	1.06
4	24.6	75.4	142	42	1.10

a All polymerizations were performed at room temperature in toluene, targeting 400 repetition units and varying the DEVP/DAlVP/Catalyst ratio. Quantitative conversions were determined via ^31^P NMR spectroscopy in CD_3_OD.

b Determined via ^1^H NMR spectroscopy by comparison of the CH_2_ signals of DEVP (4.18 ppm, m = I/4) and DAlVP (4.63 ppm, *n* = I/4);

c Calculated via ^1^H NMR spectroscopy by comparison of the – OTBDMS signals of the initiator at 0.14 ppm (I = 6H) and the CH_2_ signals of DEVP (4.18 ppm, m = I/4) and DAlVP (4.63 ppm, *n* = I/4);

d Initiator efficiency: IE = *M*_n,theo_/*M*_n,NMR_ with *M*_n,NMR_ = 327.54 g/mol+m*164.14 g/mol+n*188.16 g/mol and *M*_n,theo_ determined from the applied monomer to catalyst amounts;

e Polydispersity index determined via size-exclusion chromatography multiangle light scattering (SEC-MALS) in THF:H_2_O (1:1) with 340 mg/L 2,6-di-*tert*-butyl-4-methylphenol (BHT) and 9 g/L tetra-*n*-butylammonium bromide (TBAB).

As described in previous studies, these results highlight the high precision of REM-GTP in terms of tailoring the copolymer microstructure toward different DEVP/DAlVP ratios by adjusting the amounts of monomers relative to the catalyst while maintaining narrow polydispersities.^37^Table 1 displays a variation of the content of cross-linkable DAlVP, simultaneously targeting comparable molecular weights between 127 and 142 kg/mol in copolymers 1–4. The results for the copolymer microstructures and molecular weights were obtained via ^1^H NMR spectroscopy (Figure S1), and ^31^P NMR spectroscopy (Figure S2) and ^1^H DOSY NMR spectra (Figure S3) confirm successful copolymer synthesis. Furthermore, the polydispersities below 1.12 for entries 1–4 determined by SEC-MALS reflect a uniform molecular weight determination characteristic of catalytic REM-GTP. In a subsequent step, P(DEVP-*stat*-DAlVP) polymers 1–3 were subjected to side chain modification with 0.30 equiv of TMSBr, whereas polymer 4 was reacted with only 0.15 equiv of TMSBr (Scheme 1, Step 3). This polymer-analogous transformation is well-known for poly(vinylphosphonates), resulting in the formation of vinylphosphonic acid (VPA) units in the polymer backbone while maintaining the degree of polymerization.^43^ After purification via dialysis against deionized water (MWCO = 8 kDa), lyophilization yielded pure P(DEVP-*stat*-DAlVP-*stat*-VPA) terpolymers, in which a statistical distribution of monomers is assumed. The degree of deprotection after each reaction, also reflected by the number of VPA units formed, was calculated via comparison of the ^1^H NMR spectra of P(DEVP-*stat*-DAlVP) (Table 1, Entries 1–4) and P(DEVP-*stat*-DAlVP-*stat*-VPA) (Table 2, Entries 5–8), normalizing the signals of the polymer backbone (δ = 1.65–3.00 ppm). Notably, the side-chain hydrolysis of copolymers 1–4 (Table 1) toward terpolymers 5–8 (Table 2) resulted in a preference for DAlVP dealkylation compared to DEVP, as evidenced via ^1^H NMR spectroscopy (Figure S4) and the compositions listed in Table 2. In this context, we hypothesize that this might be due to the proposed mechanism presented in Figure S4, agreeing with the literature and suggesting an allyl cleavage rather than ethyl cleavage for statistical reasons (different reaction pathways indicated by arrows) and due to better stabilization of the corresponding cation.^1,46^ In general, however, the compositions of P(DEVP-*stat*-DAlVP-*stat*-VPA) terpolymers 5–7 presented in Table 2 suggest similar overall degrees of dealkylation, as evidenced by the extent of VPA formed, which aligns well with the application of equimolar amounts of TMSBr. Therefore, it can be concluded that controlling the amount of residual DAlVP units in the terpolymers available for cross-linking is only possible by adjusting the DEVP/DAlVP ratio during copolymer synthesis.

**Table 2:** Calculated Compositions of P(DEVP-stat-DAlVP-stat-VPA) Terpolymers Obtained through Polymer-Analogous Transformation of P(DEVP-stat-DAlVP) with TMSBr and Water Uptake of the Corresponding Hydrogels, Followed by a Description of the Mechanical Properties of the Water-Swollen Hydrogel Samples

P(DEVP-*stat*-DAlVP-*stat*-VPA)	DEVP [%]^a^	DAlVP [%]^a^	VPA [%]^a^	Water uptake of corresponding hydrogel [g (H_2_O)/g (HG)]^b^	Description of mechanical properties^c^
5	72.7	0.50	26.8	150 ± 27	No structural integrity
6	72.0	3.70	24.3	29 ± 4	Soft and brittle
7	65.6	6.80	27.6	28 ± 5	Stable specimen
8	72.1	8.20	19.7	19 ± 2	Stable specimen

a Polymer composition of functionalized samples after purification as determined from ^1^H NMR spectroscopy.

b Water uptake of corresponding hydrogels with standard deviation: *Q* = (*M*_s_ – *M*_d_)/*M*_d_.

c Obtained from the handling of the water-swollen hydrogel samples during the swelling experiments.

### Hydrogel Synthesis and Characterization of Swelling Properties

The purified P(DEVP-stat-DAlVP-stat-VPA) terpolymers were subsequently subjected to a thiol–ene click reaction with 3,6-dioxa-1,8-octanedithiol using photochemical reaction conditions (λ = 365 nm) and 2,2-dimethoxy-2-phenylacetophenone (DMPA) as a photoinitiator (Scheme 1, Step 4). Each sample was cross-linked in a UV reactor for 60 min and dried to weight constancy in vacuo. After that, each dry sample was immersed in water for eight hours, and the water uptake was calculated by comparing the weight difference between the swollen and the dry sample according to Equation S1. In previous studies, P(DEVP-stat-DAlVP) copolymers were cross-linked without further modification, yielding relatively apolar materials with water uptakes of up to 2.22 g of water per gram of dry hydrogel. A hydrophilicity increase was possible by polymer modification via thiol–ene click chemistry with sodium 3-mercaptopropane-1-sulfonate before cross-linking, resulting in swelling ratios above 50. Here, we explored a different approach toward highly polar, cross-linked materials with the beneficial side-effect of introducing stimuli-responsiveness into these novel hydrogels.^37^ The terpolymer compositions calculated via ^1^H NMR spectroscopy (Figure S4), the results of the swelling experiments, as well as a description of the mechanical properties of the hydrogels are presented in Table 2. In this context, swelling experiments were performed at least in triplicate.

When comparing the terpolymer compositions presented in Table 2 with the initial compositions of P(DEVP-*stat*-DAlVP) 1–4 (Table 1), these results reflect the preference for DAlVP-side chain dealkylation over DEVP side chain deprotection, as discussed above and confirmed by ^1^H NMR spectroscopy (Figure S4). However, treating copolymers 1–3, which display increasing numbers of DAlVP units with equimolar equivalents of TMSBr (0.30 equiv), gave access to terpolymers 5–7, which revealed residual DAlVP contents in the same sequence. Regarding the water uptake of the corresponding hydrogels, the high values of water absorption compared to those obtained in initial studies for the cross-linking of P(DEVP-*stat*-DAlVP) indicate an overall significant increase in the hydrophilicity of the gels induced by the polymer-analogous transformation.^37^ Furthermore, relating the swelling ratio of the corresponding hydrogels to the polymer compositions, the water uptake is mainly governed by the cross-linking density given by the content of DAlVP in P(DEVP-*stat*-DAlVP-*stat*-VPA), which increases from polymer 5 to 8. This is particularly evident when polymers 5 and 6 are compared with very similar amounts of DEVP and VPA, mainly differing in the number of DAlVP units. While hydrogels from polymer 6 exhibited considerable swelling, the water absorption of polymer 5 indicated a superabsorbent material with a water uptake of about 150 g of water per gram of dry hydrogel, marking a high-performance material with significant potential, e.g., in agricultural or hygienic applications.^47−50^ Comparing hydrogels from polymers 6 and 7, both materials show similar water uptake, despite notable differences in their DAlVP content. Therefore, we believe these examples illustrate an interplay between the cross-linking density and the polarity of the polymers. More specifically, polymer 7 contained 6.80% cross-linkable units, which exceeded the number of available sites in polymer 6. Nevertheless, polymer 7 also revealed the highest amount of vinylphosphonic acid units, which should yield the most polar cross-linked network and, thus, could explain the similar values for the water uptake of samples originating from polymers 6 and 7. Considering the mechanical properties of the hydrogels, an opposing trend compared to the water uptake regarding the DAlVP content was found. Whereas the swollen hydrogels arising from polymers 5 and 6 exhibited no or only poor mechanical stability, the specimen synthesized from polymers 7 and 8 remained mechanically stable in the swollen state and could be gently handled with tweezers (Figure S6). Apart from the description of the mechanical properties of the swollen states, exemplary frequency sweeps conducted on hydrogels originating from polymers 6 and 8 accentuate these findings. In both cases, the hydrogels were formed from water between the rheometer plates in situ. The higher values of the storage and loss modulus of the more densely cross-linked polymer networks indicate a higher mechanical strength of the swollen specimen (Figure S7), aligning well with the findings from previous studies.^37^

### Investigations on the pH-Responsiveness of Thin Films

**Figure 1:**
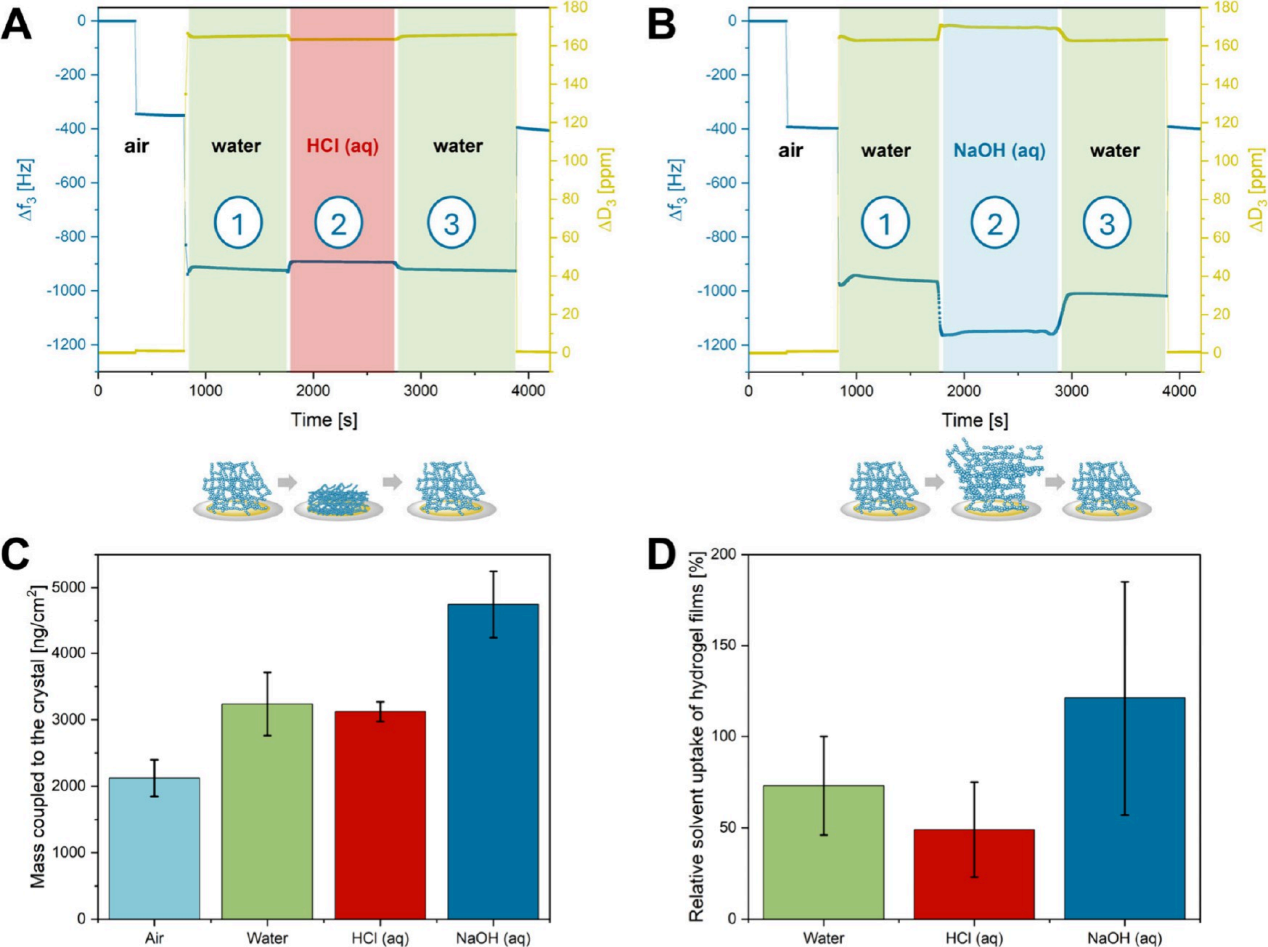
QCM-D measurements of thin films of cross-linked P(DEVP-*stat*-DAlVP-*stat*-VPA) in different aqueous media and measurements in ambient air before and after exposure to the liquids, checking for potential leaching of samples. (A) Investigation of sample behavior under acidic conditions (pH 1) and (B) investigation of sample behavior under alkaline conditions (pH 13). (C) Coupled hydrogel film masses in different environmental conditions obtained from the frequency changes of the third overtone and by subtracting the frequency decrease of each solvent on neat crystals. (D) Weight percentage of water in the films (mass of water per dry film mass) under different environmental conditions. Water masses were obtained from the coupled hydrogel film masses (water + hydrogel) by subtracting the masses obtained through the frequency decrease upon dry film deposition on the QCM-D sensors.

After investigating the water uptake of these novel P(DEVP-*stat*-DAlVP-*stat*-VPA)-based hydrogels, we aimed to understand their behavior in media with pH values other than water since the introduction of vinylphosphonic acid units should induce pH-responsive behavior. For this purpose, thin films of cross-linked, partially hydrolyzed poly(vinylphosphonates) were prepared on different substrates by applying a well-established spin-coating procedure reported for various biomaterials.^44,45^ In this context, a less than 0.01 wt % solution of polymer 8 in methanol/water (6/1) was applied to obtain thin hydrogel films on either silicon wafers or the Au electrodes of quartz-crystal microbalance (QCM-D) sensors. The processing of poly(vinylphosphonate)-based hydrogels by spin-coating has not been described before, and the fabrication of thin films of these materials could be relevant for various applications. The dry films of cross-linked, partially hydrolyzed poly(vinylphosphonates) were investigated by profilometry, light microscopy, QCM-D, and AFM. Characterization of the thin films by profilometry on cleaned silicon wafers revealed an average layer thickness of 39.4 ± 2.33 nm and a roughness average of 1.96 ± 0.75 nm determined from 12 measurements on 3 different substrates (Table S1), indicating relatively smooth and homogeneous coatings. An exemplary profilometry measurement is shown in Figure S8. Furthermore, the dry film mass was obtained from QCM-D measurements of crystals before and after spin coating and irradiation of the polymer film deposited on the quartz crystal resonator. Whereas the resonance frequency *f* decreased when the hydrogel was introduced, the dissipation factor *D*, which is the second measure of QCM-D and reflects the viscoelastic properties of the sample, increased simultaneously. This is due to the deposition of the hydrogel, resulting in an energy loss of the oscillating system due to its viscoelasticity. Given the frequency shifts of the third overtone upon spin coating, the mass coverage on the Au-surface was calculated using the Sauerbrey eq (Equation S2). Applying the average resonance frequency change *Δf*_3_ of −360 ± 46 Hz obtained from 12 QCM-D measurements, the resulting mass per area on the resonators was calculated to be 2.12 ± 0.27 μg/cm^2^. Finally, atomic force microscopy was applied to further explore the surface morphology of the hydrogels spin-coated onto the QCM-D sensor (Figure 3A). AFM indicated quantitative surface coverage of the crystals. However, the AFM images along with the recordings of the light microscope (Figure S9) revealed minor inhomogeneities on the substrate surfaces. This could either arise from a nonperfectly dissolved polymer in the spin coating solution, which was not assessable with the naked eye, or some unintended self-assembly processes on the surfaces. Nevertheless, we studied the behavior of these thin films of cross-linked P(DEVP-*stat*-DAlVP-*stat*-VPA) via QCM-D to gain insight into the pH-responsive behavior of the novel materials. First, a baseline corresponding to the resonance frequency of an uncoated sensor and, therefore, to *Δf*_3_ = 0 Hz was established in ambient air. Subsequently, the same sensor was spin-coated with an 85 ppm (0.0085 wt %) solution of polymer 8 (Table 2), containing the cross-linker and photoinitiator, and irradiated (λ = 365 nm, *t* = 1 min) to yield a cross-linked network on the Au-coated surface of the QCM-D sensor. Following this step, remeasurement of the crystals in air confirmed successful spin coating by a frequency change of −360 ± 46 Hz, which is also observable in the first decrease in *Δf*_3_ highlighted in Figures 1A and 1B.

**Figure 2:**
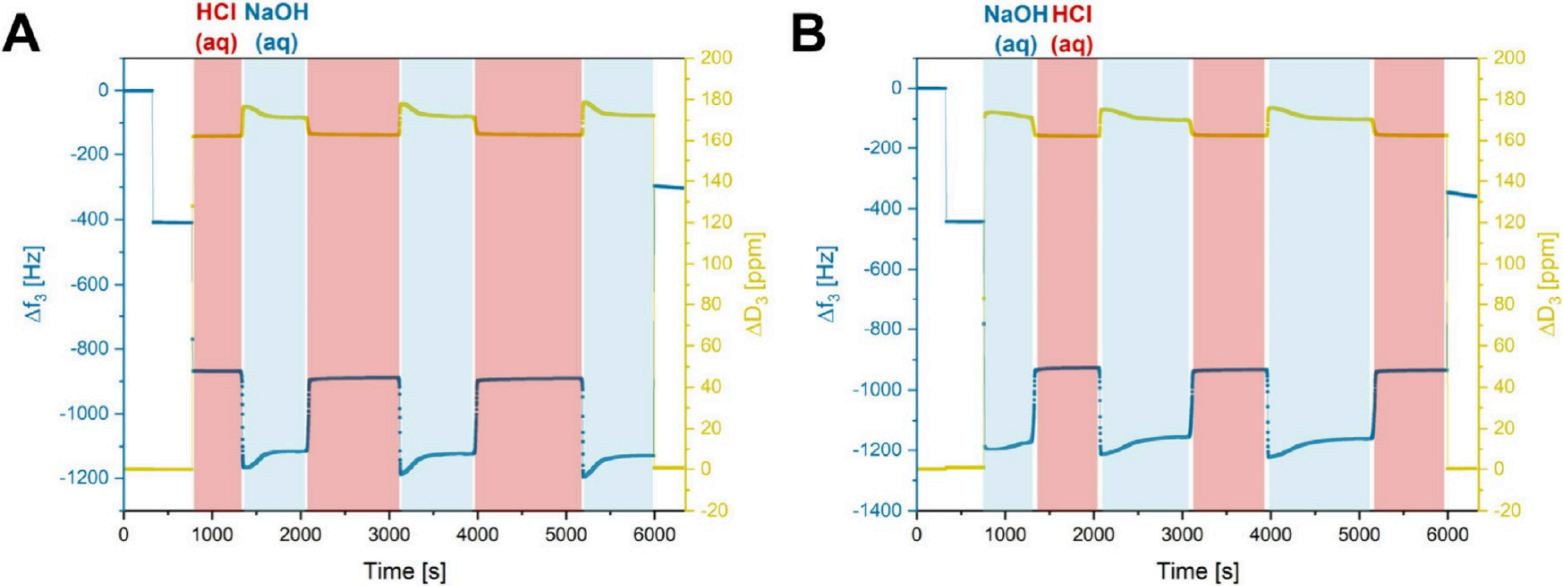
QCM-D measurements of thin films of cross-linked P(DEVP-*stat*-DAlVP-*stat*-VPA) in alternating aqueous media (two pH values) and measurements on air before and after exposure to the liquids. (A) Cycling of the pH value between pH 1 (0.1 M HCl solution) and pH 13 (0.1 M NaOH solution) over three cycles. (B) Cycling of the pH value between pH 13 (0.1 M NaOH solution) and pH 1 (0.1 M HCl solution) over three cycles.

Transitioning from air to water, both measurements in Figure 1 reveal a significant decrease in the oscillation frequency of the third overtone of the crystals, dropping to frequencies around *Δf*_3_ = −940 ± 80 Hz, which is a commonly observed phenomenon due to density changes in the medium. However, a substantial contribution toward this frequency difference is caused by the swelling of the hydrogel film on the QCM-D resonator. Subtracting the frequency changes through dry film deposition (*Δf*_3_ = −360 ± 46 Hz) and water on an empty QCM-D crystal (*Δf*_3_ = −392 Hz, Figure S10) would indicate a weight increase through hydrodynamically coupled water of roughly 1.11 μg/cm^2^ as determined by the Sauerbrey equation, disregarding that this relation becomes nonlinear in swollen systems without full elastic coupling to the sensor. Once stable QCM-D signals were reached, indicating the equilibrium swelling state of the hydrogel films, the aqueous environments were altered to either 0.1 M hydrochloric acid (aq) (pH 1) or 0.1 M sodium hydroxide solution (aq) (pH 13), while the swelling behavior of each film. Switching to acidic measurement conditions (Figure 1A), a sharp frequency increase to *Δf*_3_ = −923 ± 25 Hz, along with a decrease of the dissipation to *ΔD*_3_ = 164 ± 1 ppm, was observed. Both trends can be explained by a collapse of the formerly partially deprotonated hydrogel at pH 7 through protonation at pH 1 and, therefore, the loss of electrostatic repulsion. This hypothesis is supported by the titration curve of PVPA (Figure S5), revealing a gradual increase of the pH value, indicating a dynamic deprotonation/protonation of the polymers, and suggesting that parts of the PVPA might as well be deprotonated at a pH value of 7. Furthermore, the decrease in dissipation is explained by the lower viscoelasticity of the swollen hydrogel upon collapse, causing a smaller energy loss than in the initial state. The exact opposite trend in the frequency and dissipation values is observed for alkaline conditions (Figure 1B). Upon displacement of distilled water by NaOH (aq) in the measurement cell, a sharp decrease in the frequency to *Δf*_3_ = −1195 ± 85 Hz and an increase in the dissipation to *ΔD*_3_ = 170 ± 2 ppm, followed by plateaus of either, was detected. In analogy to the explanation above, this, in turn, is accounted for by an extensive swelling of the hydrogel network due to electrostatic repulsion of the deprotonated vinylphosphonic acid units in the polymers, resulting in a higher mass of water coupled to the QCM-D sensor. With the aid of the titration curve of PVPA (Figure S5), displaying the behavior of a monoprotic acid and a gradual deprotonation, it becomes obvious that under the harsh conditions of pH 13, large parts of the VPA units in cross-linked P(DEVP-*stat*-DAlVP-*stat*-VPA) should be deprotonated. The strong electrostatic repulsion explains the more drastic changes in *Δf*_3_ and *ΔD*_3_ switching from pH 7 (partially deprotonated) to 13 (mostly deprotonated) than that from pH 7 (partially deprotonated) to 1 (mostly protonated). This trend is also reflected in the calculated coupled masses (hydrogel + water) in different environments (Figure 1C) and can also be seen in Figure 1D, which shows the mass of water per dry film mass for the different swelling states. Comparing the water uptake found in the QCM-D studies for thin films of cross-linked polymer 8 (Figure 1D) with the water absorption of the macromolecular objects (Table 2), a significant reduction in hydrogel swelling can be observed. This could be attributed to the restrained chain mobility of the polymer chains due to the surface attachment, making the polymers more immobile for swelling and hindering water accessibility into the networks. Nevertheless, the results in Figure 1D reveal pH-dependent swelling of the thin hydrogel film. Notably, control experiments confirmed that the observed frequency and dissipation changes in aqueous environments, shown in Figure 1, arise exclusively from the film behavior on the QCM-D sensors since the density or other solvent effects had no impact on the values of frequency and dissipation when measuring uncoated sensors (Figure S10). Next, the reversibility toward the initial swelling state in water was confirmed by returning to pH 7 in both cases. This resulted in plateaus of the signals and yielded similar data for *Δf*_3_ and *ΔD*_3_ as initially determined, therefore not hinting toward any mass loss on the sensors. To confirm this assumption, the measurements were stopped and the crystals dried thoroughly using nitrogen gas. Remeasurement of the dry crystals (final plateaus in Figures 1A and 1B) and comparison with the pristine masses confirmed that no detachment of the hydrogel films from the Au surfaces of the QCM-D resonators occurred despite exposing the films to the harsh conditions discussed above. To further explore the application potential of these materials in devices, the reversibility of swelling and collapse of thin films of P(DEVP-*stat*-DAlVP-*stat*-VPA)-based hydrogels at these extreme pH values was studied over the course of three cycles (Figures 2A and 2B).

**Figure 3:**
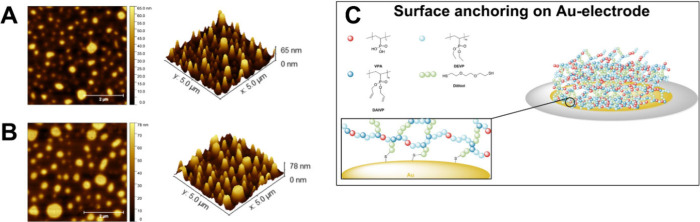
AFM images of the native hydrogel on the Au-surface of a QCM-D crystal (A), the hydrogel after QCM-D measurements with alternating pH values and subsequent drying of the crystals (B), and proposed surface anchoring of P(DEVP-*stat*-DAlVP-*stat*-VPA)-based hydrogels by the dithiol cross-linker via interaction with the gold electrode (C).

In these experiments, QCM-D sensors spin-coated with a P(DEVP-*stat*-DAlVP-*stat*-VPA)-containing solution and subsequently cross-linked via UV irradiation were first investigated in air, resulting in the frequency and dissipation changes upon mass deposition mentioned previously. When exposing the films on the crystals to acidic conditions (pH 1), *Δf*_3_ = −923 ± 25 Hz and *ΔD*_3_ = 164 ± 1 ppm were obtained (Figure 2A). Switching to alkaline conditions (pH 13) after reaching stable measurement values, a sharp decrease in the frequency to *Δf*_3_ = −1195 ± 85 Hz and an increase in the dissipation to *ΔD*_3_ = 170 ± 2 ppm was obtained, indicating extensive swelling of the hydrogel film as discussed above. These values were successfully reproduced when performing this solvent exchange for two subsequent cycles, indicating fully reversible swelling and deswelling of the film without detachment during the online monitoring of the *f* and *D* values. The opposite behavior was observed when starting the measurements at high pH values (Figure 2B). To confirm the preservation of the hydrogel layers on the QCM resonators, the crystals were dried and remeasured in air, yielding comparable data of *Δf*_3_ and *ΔD*_3_ as opposed to their initial values, suggesting no significant weight loss after three cycles. To further substantiate this assumption, AFM measurements of the coated sensors after QCM-D cycling experiments were conducted to investigate the surface topography. Comparing the AFM measurements before (Figure 3A) and after (Figure 3B), the QCM-D experiments revealed similar surface coverages of the hydrogel and did not hint toward any film loss caused by the harsh conditions. The excellent adhesion of the films on the gold substrates led to the hypothesis that the dithiol cross-linker applied in hydrogel synthesis might act as a surface anchor interconnecting the hydrogel network with the electrode surface (Figure 3C). This hypothesis is supported by numerous reports on the interaction thiol- or sulfur-containing polymers with Au electrodes in QCM-D,^51−53^ and by control experiments, in which exposing films of non-cross-linked polymers to different aqueous environments resulted in a significant sample loss (Figure S11).

**Figure 4:**
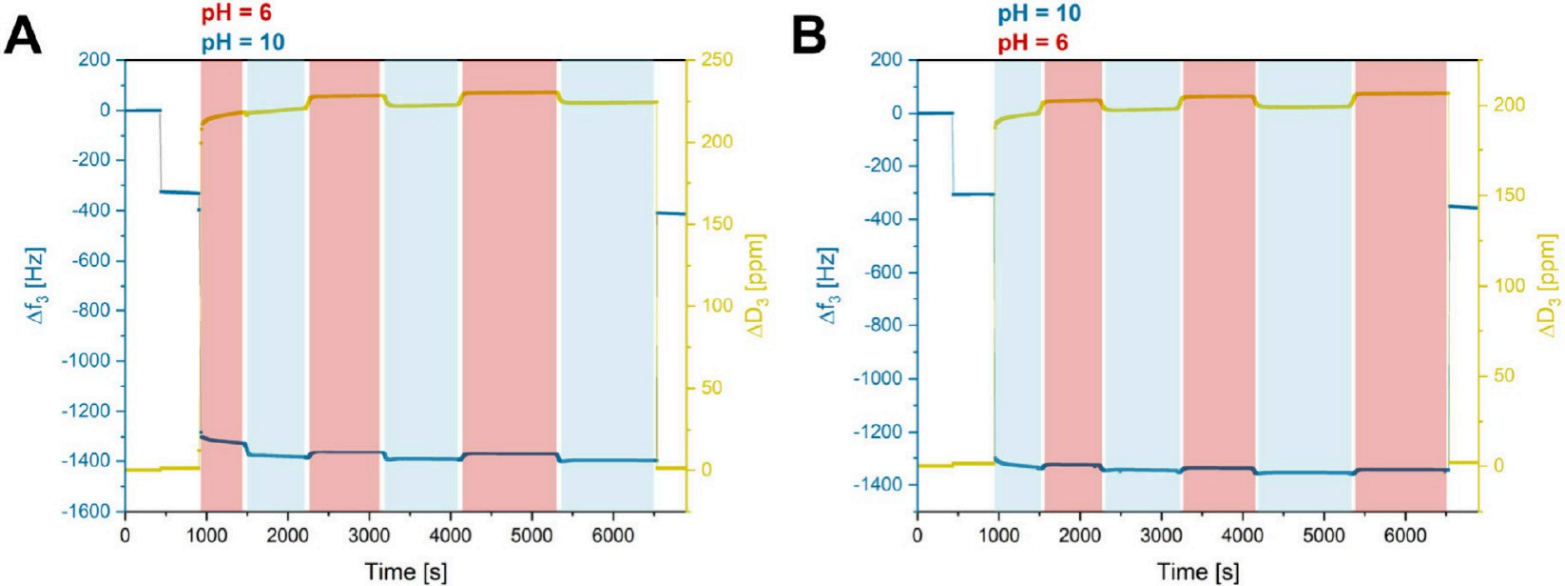
QCM-D measurements of thin films of cross-linked P(DEVP-*stat*-DAlVP-*stat*-VPA) in different buffers and measurements in air before and after exposure to the liquids. (A) Cycling of the pH value between pH 6 (0.1 M citrate buffer) and pH 10 (0.1 M carbonate buffer) over three cycles. (B) Cycling of the pH value between pH 10 (0.1 M carbonate buffer) and pH 6 (0.1 M citrate buffer) over three cycles.

To demonstrate the broader applicability of these novel materials, the pH-responsiveness was studied under milder conditions (2 pH units above and below the equivalence point of the titration of PVPA with NaOH, Figure S5) by investigating the thin film behavior of cross-linked P(DEVP-*stat*-DAlVP-*stat*-VPA) in 0.1 M citrate buffer (pH 6) and 0.1 M carbonate buffer (pH 10) (Figures 4A and 4B). Again, the *Δf*_3_ values obtained in these experiments demonstrate high reversibility between the swelling states over three cycles regardless of the order in which the buffers were added to the samples. Each experiment was performed in triplicate, revealing excellent reproducibility and yielding *Δf*_3_ = −1395 ± 47 Hz for the collapsed state (pH 6) and *Δf*_3_ = −1427 ± 38 Hz for the swollen state (pH 10), allowing differentiation between the two pH values. The higher frequency decreases compared to the measurements presented in Figure 3 might hint toward buffer interactions with the films or density effects. Unlike in the case of HCl (aq) and NaOH (aq) (Figure S10), measurements of uncoated QCM-D crystals in the buffered solutions indeed showed a dependence of the resonance frequency and the dissipation on the surrounding medium (Figure S12). In this context, the frequency increased switching to the carbonate buffer, counteracting the swelling-induced frequency decrease, and vice versa for the citrate buffer. Consequently, the frequency window for pH differentiation might effectively appear smaller than that for other solutions in the same pH window. In contrast to the measurements with NaOH (aq) and HCl (aq), the values for *ΔD*_3_ do not inversely correlate with the frequency states but seem to be dominated by the viscoelastic properties of the buffers as evidenced by the corresponding control experiments (Figure S12).

Nevertheless, the results presented in Figures 2 and 4 reveal the pH-responsive behavior of cross-linked P(DEVP-*stat*-DAlVP-*stat*-VPA) polymers in different media, suggesting a high potential for implementing the material properties in various applications. In this context, the excellent adhesion of hydrogel films on gold surfaces combined with the photochemical cross-linking process allows photolithographic micropatterning, which is relevant not only in the biomedical field but also in optics and electronics.^54,55^ Further, the extensive swelling of these hydrogels; their ability to form thin, homogeneous films; and their pH-dependent volume changes render them ideal candidates for developing piezoelectric pH-sensors.^56,57^ For device fabrication, a more detailed study of the correlation between the water uptake and the film thickness is crucial, as the substrate polarity can dominate the swelling properties for very thin films.^58,59^ Very thin films further comprise layers with less mobile polymer chains due to the interaction with the solid Au substrate, leading to fewer degrees of freedom and therefore to less swelling and a lower relative mass increase. A fundamental understanding of this dependency, in turn, might help to adjust the sensor’s sensitivity in future studies.

### Surface Analysis of Thin Films on Gold Substrates

**Figure 5:**
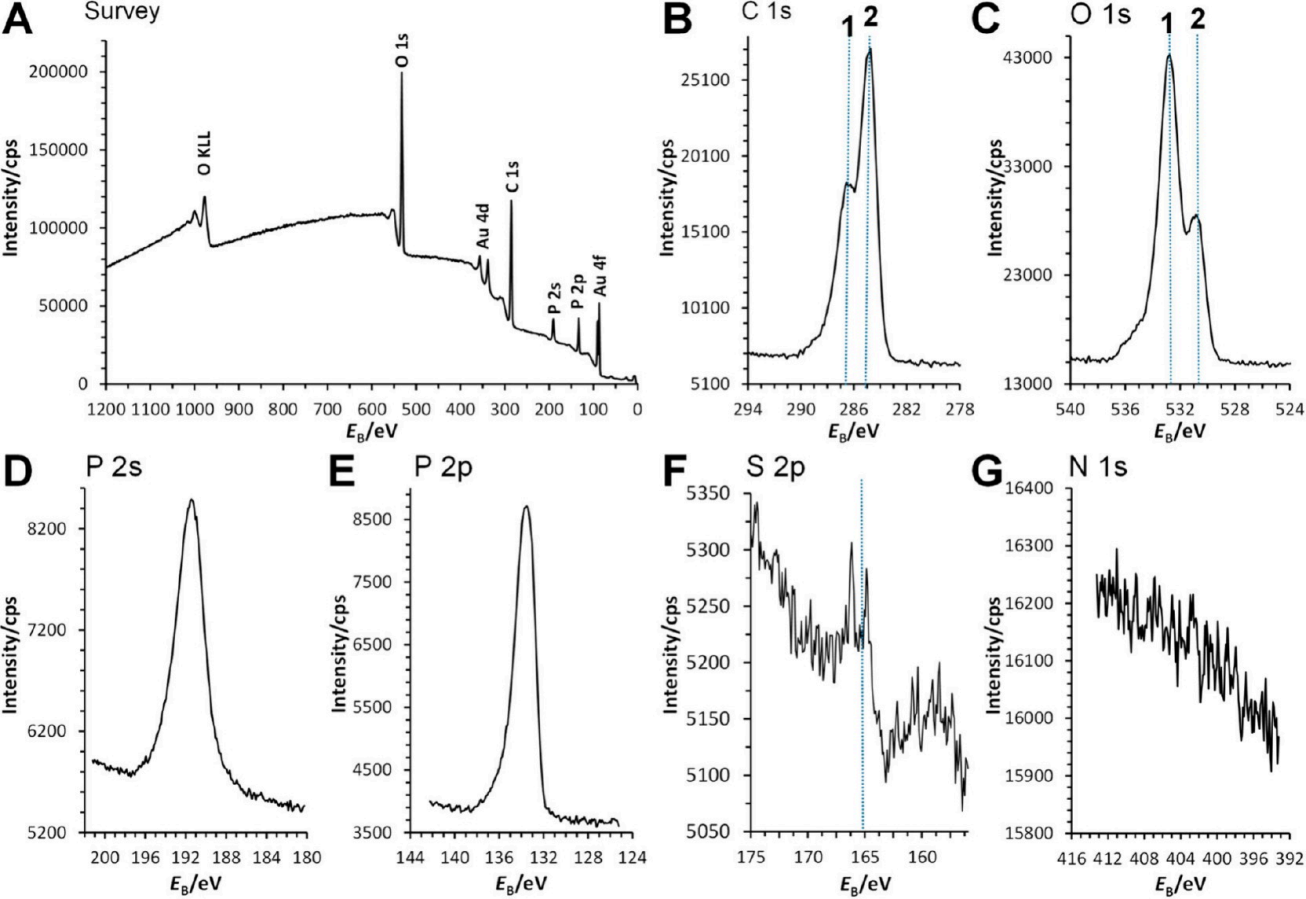
Survey (A) and high-resolution XPS spectra for (B) C 1s, (C) O 1s, (D) P 2s, (E) P 2p, (F) S 2p, and (G) N 1s.

To further elucidate the interactions between the films of cross-linked P(DEVP-*stat*-DAlVP-*stat*-VPA) polymers and the Au surfaces of the QCM-D sensors, in-depth surface analysis of the spin-coated films was conducted via X-ray photoelectron spectroscopy (XPS) and time-of-flight secondary ion mass spectrometry (ToF-SIMS). Figure 5 presents the survey and high-resolution XPS spectra, providing an overview of the surface composition. The survey spectrum reveals the presence of O-, C-, Au-, and P-containing species on the surface. However, the S- and N-related signals, which could confirm the presence of the dithiol cross-linker and the polymer end-group, were not developed in the XPS spectra (Figures 5A, 5F, 5G).

**Figure 6:**
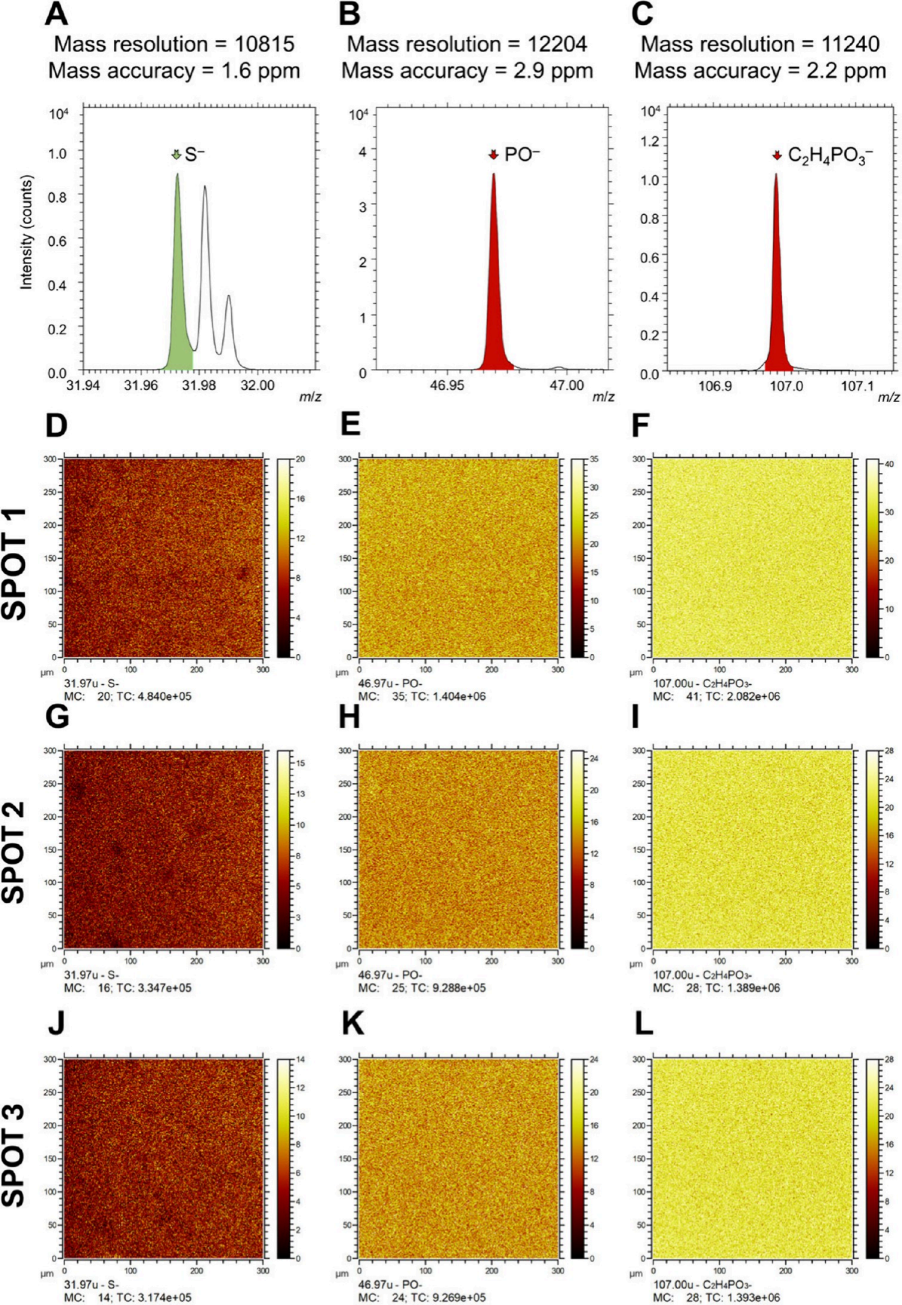
Negative ion ToF-SIMS spectra showing the peaks for (A) S^–^, (B) PO^–^, and (C) C_2_H_4_PO_4_^–^, along with corresponding two-dimensional ToF-SIMS images for spot 1 (D–F), spot 2 (G–I), and spot 3 (J–L) on the surface, illustrating the spatial distribution of S^–^, PO^–^, and C_2_H_4_PO_4_^–^ signals.

**Figure 7:**
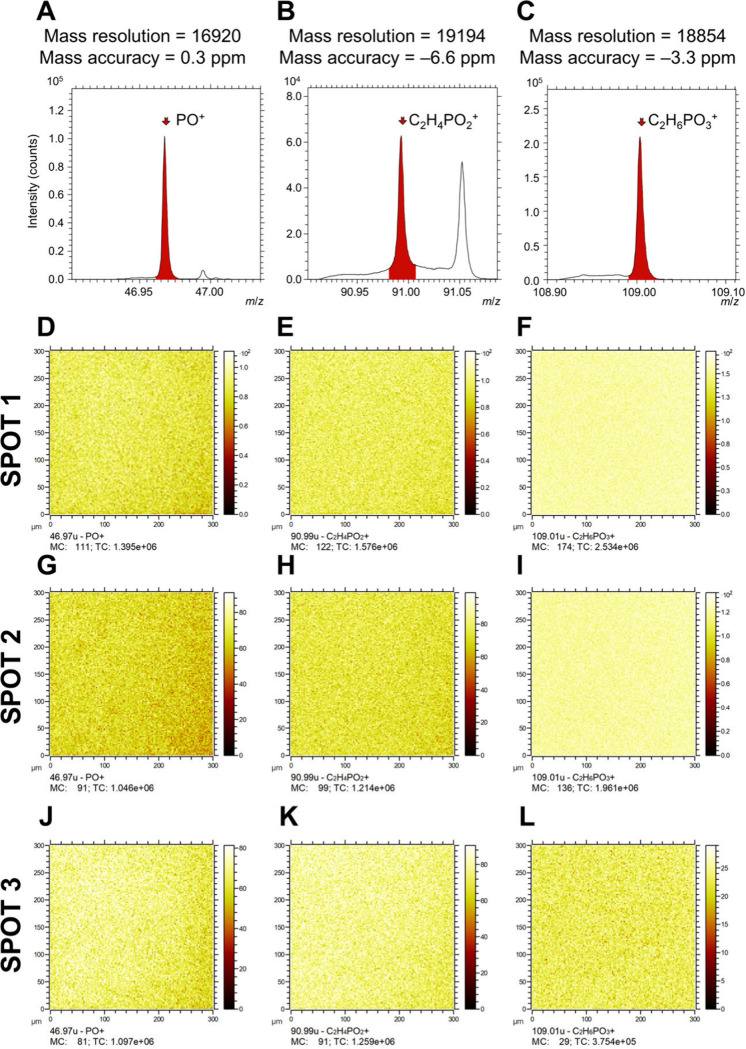
Positive ion ToF-SIMS spectra showing the peaks for (A) PO^+^, (B) C_2_H_4_PO_3_^+^, and (C) C_2_H_6_PO_3_^+^, along with corresponding two-dimensional ToF-SIMS images for spot 1 (D–F), spot 2 (G–I), and spot 3 (J–L) on the surface, illustrating the spatial distribution of PO^+^, C_2_H_4_PO_3_^+^, and C_2_H_6_PO_3_^+^ signals.

**Figure 8:**
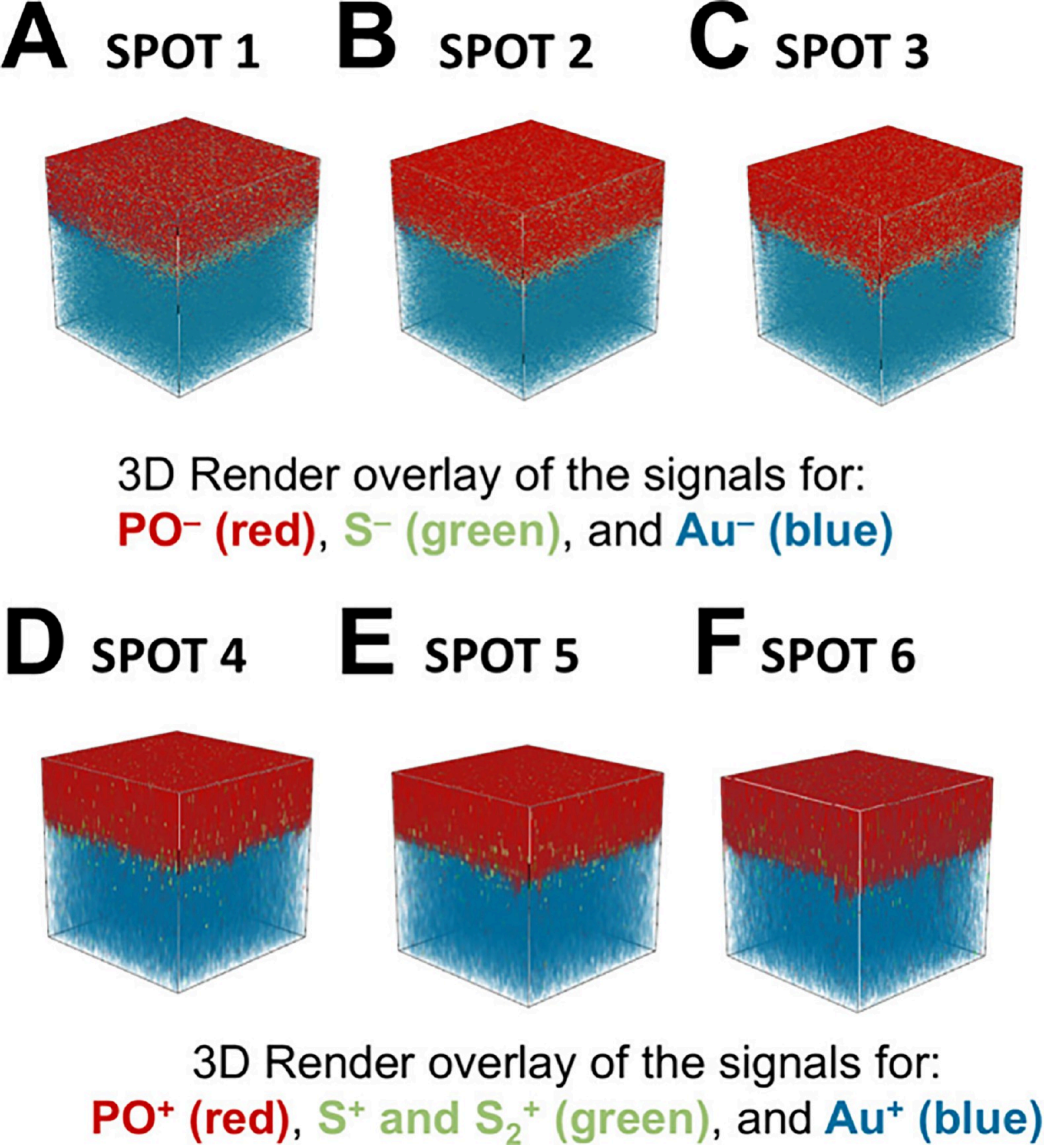
Three-dimensional ToF-SIMS images for 6 different spots of the surface showing the distribution of PO^–^, S^–^, Au^–^ signals (A–C), and PO^+^, S^+^, S_2_^+^, and Au^+^ signals (D–F). Sputtering was performed by using 2.5 keV Ar_1300_^+^. *x*- and *y*-scales are 300 μm × 300 μm.

**Figure 9:**
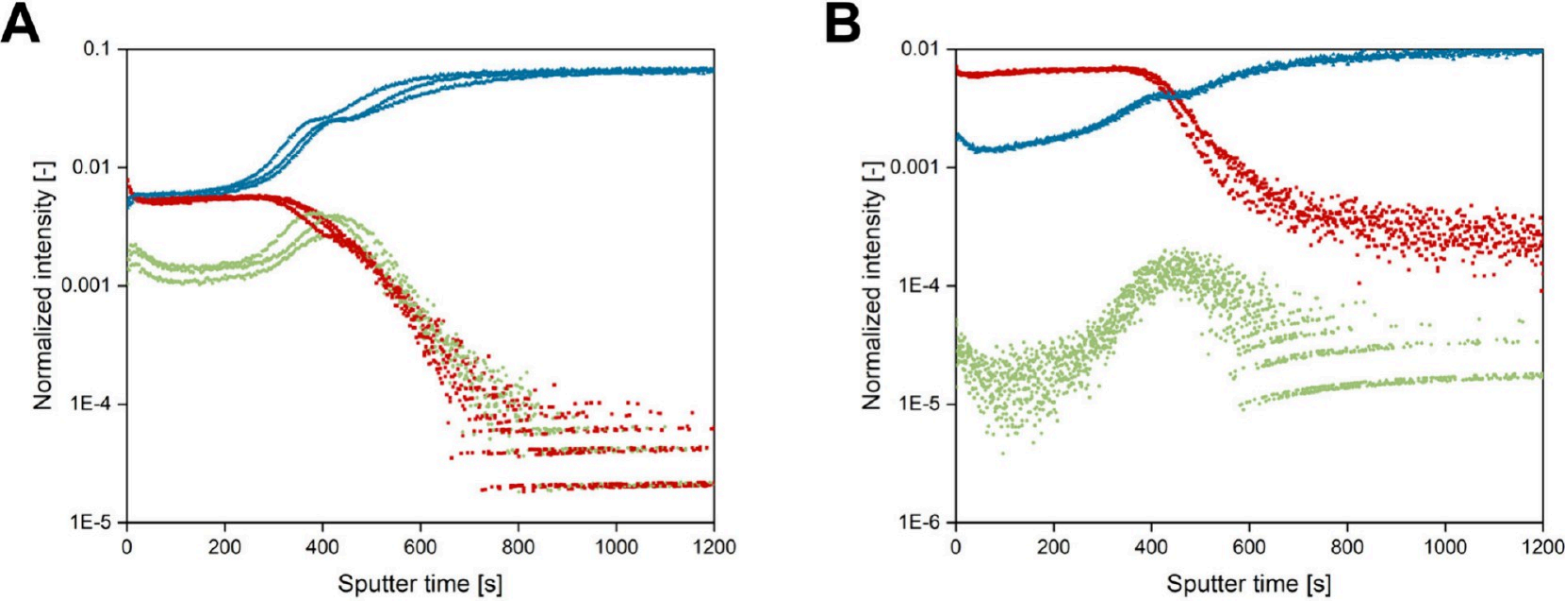
Depth profiles measured at three different spots on the surface in (A) negative polarity (Au^–^ in blue, PO^–^ in red, and S^–^ in green) and (B) positive polarity (Au^+^ in blue, PO^+^ in red, and S^+^/S_2_^+^ in green). Normalization was applied based on the total ion intensities.

Figure 5B displays the C 1s spectrum, which clearly identifies C–C/C–H containing species at 284.8 eV (dashed line 2) and C–O containing species at a distinct binding energy (dashed line 1). The O 1s spectrum (Figure 5C) indicates two distinct oxygen environments, marked at dashed lines 1 and 2, which could correspond to R-PO_3_-R_2_ and R-PO_3_H_2_ groups, respectively, consistent with the polymer’s expected structure. The phosphorus signals are intense in the XPS spectra, as shown in Figures 5A, 5D, and 5E. The P 2p spectrum, in particular, confirms the presence of R-PO_3_-R_2_ moieties, as evidenced by the binding energy (*E*_B_) position. This observation supports the conclusion that the polymer is present on the Au surface. The S 2p and N 1s spectra (Figures 5F and 5G) show no detectable signals, indicating that the sulfur and nitrogen concentrations are too low for this technique. While a peak begins to emerge in the S 2p spectrum (Figure 5F), its intensity remains below the threshold for confident detection, as it does not exceed three times the background noise. Sulfur should, however, be incorporated into the hydrogel structure due to the cross-linker. N-containing species, in turn, might originate from the polymer end groups. The limitations of XPS were addressed using ToF-SIMS, which has a lower detection limit and successfully confirmed the presence of S-containing species, providing a more comprehensive surface characterization. The cross-linked films on the surfaces comprise R-PO_3_-containing moieties (R being the aliphatic polymer backbone) and S-containing moieties. Using ToF-SIMS, the polymer was characterized by S^–^, PO^–^ and C_2_H_4_PO_3_^–^ signals in negative polarity (Figures 6A–C) and PO^+^, C_2_H_4_PO_3_^+^, and C_2_H_6_PO_3_^+^ signals in positive polarity (Figures 7A–C). These signals provided high mass resolution and accuracy, as designated in these figures. Two-dimensional ToF-SIMS imaging based on these signals revealed the homogeneous distribution of the cross-linked polymers on the Au electrode of the QCM sensor surface (Figures 6D–L and 7D–L). Subsequent sputtering with a 2.5 keV Ar_1300_^+^ beam gradually removed the organic material, allowing the creation of three-dimensional images (Figure 8) and depth profiles (Figure 9). The S^–^ and PO^–^ signals demonstrated the polymer distribution in negative polarity, while the S^+^, S_2_^+^, and PO^+^ signals were utilized in positive polarity. As expected, S^+^ and S_2_^+^ signals were less intense in the positive polarity. Au^–^ and Au^+^ signals served to identify the Au substrate in negative and positive polarities, respectively. Both the three-dimensional ToF-SIMS images (Figure 8) and the depth profiles (Figure 9) confirmed the accumulation of sulfur atoms at the Au surface, as evidenced by increased S-related signal intensity at the polymer network/Au interface, supporting the hypothesis of surface anchoring through Au-thiol interactions presented in Figure 3C. The three-dimensional ToF-SIMS images in Figure 8 further reveal the presence of the cross-linked polymers on the QCM-D sensors and a homogeneous distribution of the dithiol cross-linker within the P(DEVP-*stat*-DAlVP-*stat*-VPA) networks.

## Conclusion

In this study, we introduced an innovative method to synthesize highly water-absorbing, pH-responsive hydrogels via photochemical cross-linking of allyl groups in P(DEVP-*stat*-DAlVP-*stat*-VPA). The hydrophilic and cross-linkable polymers were easily accessible by polymer-analogous hydrolysis of P(DEVP-*stat*-DAlVP) copolymers obtained via highly controlled catalytic polymerization (REM-GTP). Side-chain dealkylation of poly(vinylphosphonates) using TMSBr preferentially targeted allylic over ethyl side chains, as confirmed via ^1^H NMR spectroscopy. Photochemical cross-linking of P(DEVP-*stat*-DAlVP-*stat*-VPA) yielded superabsorbent materials with water uptakes up to 150 ± 27 g (H_2_O)/g (hydrogel) and evident structure–property relationships between the cross-linking density and swelling behavior. In the second part, thin polymer films were successfully spin-coated on silicon wafers and gold electrodes of QCM-D sensors, followed by photo-cross-linking, yielding hydrogel films with a thickness of 39.4 ± 2.33 nm characterized via profilometry, QCM-D, and AFM. The hydrogel films exhibited pronounced pH-responsive behavior in QCM-D measurements when exposed to various aqueous media, displayed by the corresponding frequency and dissipation values reflecting different swelling states of the samples. In addition, the high stability of films on the sensors under harsh conditions was explained by the surface anchoring of the hydrogels via interactions of the dithiol cross-linker with the gold surface of the electrode. In-depth XPS and ToF-SIMS studies confirmed this hypothesis and contributed to a more comprehensive surface characterization of cross-linked P(DEVP-*stat*-DAlVP-*stat*-VPA) films on gold substrates. Overall, these novel materials were successfully applied as pH sensors in QCM-D experiments, and this study revealed great potential for these hydrogels to be implemented as superabsorbent networks or in photolithographic applications due to their excellent adhesion on gold surfaces. Most importantly, however, the pH-responsive properties of P(DEVP-*stat*-DAlVP-*stat*-VPA) are currently being explored for potential utilization in pH-sensors and actuators, for which these novel stimuli-responsive materials are highly promising candidates.

## Supporting Information Available

Materials and methods; Experimental procedures; Polymer characterization; Titration curve of PVPA; Hydrogel swelling experiments; Rheological measurements; Macroscopic and microscopic images of hydrogel (films); Thin-film preparation and characterization; Calculations of the mass deposition by the Sauerbrey equation; Quartz crystal microbalance control experiments (PDF)

## ABBREVIATIONS

AFM: atomic force microscopy
DAlVP: diallyl vinylphosphonate
DEVP: diethyl vinylphosphonate
VPA: vinylphosphonic acid
REM-GTP: rare earth metal-mediated group-transfer polymerization
NMR: nuclear magnetic resonance
DMPA: 2,2-dimethoxy-2-phenylacetophenone
IE: initiator efficiency
*Đ*: polydispersity index
QCM-D: quartz crystal microbalance with dissipation monitoring
*Δf*_3_: frequency change of the third overtone of the QCM-D sensor
*ΔD*_3_: dissipation change of the third overtone of the QCM-D sensor
TMSBr: trimethylsilyl bromide
MWCO: molecular weight cutoff, r.t., room temperature
SEC-MALS: size-exclusion chromatography multiangle light scattering
ToF-SIMS: time-of-flight secondary ion mass spectrometry
XPS: X-ray photoelectron spectroscopy
