# Supplementary material for: Water Uptake, Thin-Film Characterization, and Gravimetric pH-Sensing of Poly(vinylphosphonate)-Based Hydrogels
Source: ACS Appl Mater Interfaces. 2024 Dec 20;17(1):2577–91. doi: 10.1021/acsami.4c17704 (PMC11783365; doi:10.1021/acsami.4c17704)
Supplement: Supplementary file 1 — am4c17704_si_001.pdf [file am4c17704_si_001.pdf]

Supporting Information

Water Uptake, Thin-Film Characterization, and Gravimetric pH-Sensing of Poly(vinylphosphonate)-Based Hydrogels

Anton S. Maier[1], *Matjaž Finšgar*[2], *Beatrice De Chiara*[3], *Rupert Kargl*[4,*], *Bernhard Wolfrum*[3], *Karin Stana Kleinschek*[4], *and Bernhard Rieger*[1,*]

[1]Technical University of Munich, TUM School of Natural Sciences, Department of Chemistry, WACKER-Chair of Macromolecular Chemistry, Lichtenbergstraße 4, 85748 Garching, Germany

[2]Faculty of Chemistry and Chemical Engineering, University of Maribor, 2000 Maribor, Slovenia

[3]Technical University of Munich, TUM School of Computation, Information and Technology, Munich Institute of Biomedical Engineering, Department of Electrical Engineering, Neuroelectronics, Hans-Piloty-Str. 1, 85748 Garching, Germany

[4]Graz University of Technology, Institute for Chemistry and Technology of Biobased Systems (IBioSys), Stremayrgasse 9, 8010 Graz, Austria

Corresponding Authors

* rieger@tum.de

*rupert.kargl@tugraz.at

TABLE OF CONTENTS

1. Materials and Methods 3
2. Polymer and hydrogel synthesis 9
3. Thin-film preparation and characterization 15
4. Quartz crystal microbalance measurements 17
5. References 20

1. MATERIALS AND METHODS

General Experimental

All air and moisture-sensitive compounds were prepared using standard Schlenk techniques or in a glovebox with argon (99.996 vol.-%) from Westfalen as inert gas. All glass instruments were oven-dried prior to use. Unless otherwise stated, all chemicals and solvents were purchased from Sigma-Aldrich, ABCR GmbH, or TCI Chemicals and used without further purification. Dry solvents were obtained from an MBraun MB-SPS-800 solvent purification system or by drying over activated alumina and stored over activated 3 Å molecular sieves. Deuterated solvents were purchased from Sigma-Aldrich and dried over activated 3 Å molecular sieves. The monomers diethyl vinylphosphonate (DEVP), diallyl vinylphosphonate (DAlVP), the complex $Cp_2YCH_2TMS(thf)$ and the initiator 4-(4-(((tert-butyldimethyl-silyl)oxy)methyl)phenyl)-2,6-dimethylpyridine were synthesized according to literature-known procedures.[1–3] The monomers were dried over calcium hydride and distilled prior to polymerization.

Atomic force microscopy (AFM)

The surface morphology of thin hydrogel films coated onto the gold surfaces of QCM resonators was investigated using an atomic force microscope Tosca 400 from Anton Paar (Graz, Austria). The images were scanned in tapping mode with a silicon SPM-sensor (Arrow-NCR-50) from Nanoworld (Switzerland) with a resonance frequency of 285 kHz and a force constant of 42 N/m. Image sizes of 10 µm × 10 µm, 5 µm × 5 µm, and 1 µm × 1 µm were scanned at a speed of 0.9 lines/second at room temperature. Image processing was done using the Gwyddion software.

Preparation of quartz crystal microbalance sensors

QCM 5 MHz 14 mm Cr/Au sensors were purchased from Quartz Pro AB, Sweden. Before the measurements, the QCM-D crystals were cleaned in several steps. First, the substrates were immersed in a solution containing 50 mL distilled water, 10 mL ammonia (25% in water), and 10 mL hydrogen peroxide (30% in water) and heated to 70 °C for 15 minutes. Subsequently, the crystals were dipped into distilled water for 15 minutes. After drying, the crystals were brought into contact with “piranha” solution containing H_2O_2 (30%) and H_2SO_4 (85%) in a ratio of 1:3 (v/v) for 60 seconds. Finally, the previous solution was removed by placing the QCM sensors in Milli-Q water for 15 minutes before drying them with nitrogen gas. In case of residual remains on the crystals front or backside, these were carefully removed with an acetone-soaked cotton swab, and the sensors dried again.

Cleaning of silicon wafers

Silicon wafers from Topsil (Germany) with (100) surface orientation were cut into pieces of $15 \times 15\ mm^2$ and used for spin-coating, followed by layer thickness determination via profilometry. First, the substrates were rinsed with distilled water, followed by ethanol and distilled water. Subsequently, the wafers were immersed in “piranha” solution containing H_2O_2 (30%) and H_2SO_4 (85%) in a ratio of 1:3 (v/v) for 30 minutes. After removing them from the acidic solution, the silicon plates were placed in Milli-Q water for 30 minutes. Finally, each substrate was dried with nitrogen gas and thoroughly cleaned with an acetone-soaked cotton swab.

Lyophilization

Lyophilization was performed on a VaCo 5-II-D from Zirbus Technology GmbH with a pressure of 2 mbar and a condenser temperature of -90 °C. Polymers subjected to freeze-drying were

dissolved in 1,4-dioxane or distilled water before freezing in liquid nitrogen under constant rotation.

Nuclear magnetic resonance spectroscopy (NMR)

^{1}H-NMR- and ^{31}P-NMR spectra of polymers were recorded on a Bruker AV-400HD, AV-500HD, or AV-II-500 spectrometer at 400 or 500 MHz (^{1}H) and 203 MHz (^{31}P), respectively. All chemical shifts are given in parts per million (ppm) and referenced to the residual proton signal of the respective solvent (Benzene-d6: $\delta = 7.16$ ppm, Methanol-d4: $\delta = 3.31$ ppm). Deuterated solvents were purchased from Sigma-Aldrich or Deutero and dried over activated 3 Å molecular sieves. The NMR spectra were analyzed using the MestReNova software. ^{1}H DOSY NMR experiments were performed to characterize copolymers and functionalized copolymers.

Oscillatory Rheology

Rheological characterizations were performed on an MCR 302 rheometer from Anton Paar with an upper plate (25 mm diameter), a glass plate as a counterpart, and a gap size of 0.5 mm. The samples were applied in a liquid state (250 µL) and tempered to 25 °C through an upper and lower Peltier system. Additionally, a protective hood was used. A MAX-302 lamp from Asahi Spectra with a cutoff wavelength below 400 nm was used for irradiation through the bottom glass plate to form the hydrogels in situ before the measurements. Data acquisition took place every 10 seconds, and the data was monitored via the Rheoplus software. Frequency sweeps were performed at 1% deformation between 0.01 and 10 Hz.

Profilometry

The thickness of hydrogel thin films on cleaned silicon wafers was determined via profilometry using a DektakXT from Bruker. Therefore, the scan profile was set to hills and valleys with a scan length of 2000 µm in 10 seconds. Further, the Stylus radius was 12.5 µm with a force of 3 mg and a resolution of 0.666 µm/pt. Before analyzing the height profile, each sample was scratched to the substrate surface multiple times with a small razor blade to remove the films and obtain film heights with a step-height profile. In this context, film thickness determinations were carried out with 4 different substrates and at various positions on each substrate to calculate standard deviations.

Quartz crystal microbalance with dissipation monitoring (QCM-D)

QCM-D measurements were performed on cleaned quartz crystals on a QCM-D model E4 instrument from Q-Sense (Gothenburg, Sweden). The instrument simultaneously detects changes in the resonance frequency (Δf) and the energy dissipation (ΔD) caused by mass deposition or removal on an oscillating piezoelectric crystal. In this context, ΔD is applied to describe the viscoelastic properties of the material on the crystal as it refers to energy losses due to friction, causing a damping of the oscillation. Mass depositions on the crystals were calculated using the Sauerbrey equation, which correlates frequency changes Δf_n of certain overtones to the mass deposition on the crystals. A more detailed mathematical description can be found below.

Size-exclusion chromatography multi-angle light scattering (SEC-MALS)

Polydispersities of the polymers were determined via size-exclusion chromatography (SEC) with sample concentrations of 4 mg mL^{-1} on two PL Polargel-M columns (Agilent) at 40 °C. A mixture of water and THF (1:1), treated with tetrabutylammonium bromide (9 g L^{-1}) and 3,5-di-tert-butyl-

4-hydroxytoluene (340 mg L^{-1}) was used as eluent. Samples were analyzed using a Wyatt Dawn Heleos II light scattering unit in combination with a Wyatt Optilab rEX as RI detector unit.

Spin-coating of quartz crystals and silicon wafers

The spin-coating of quartz crystals and silicon wafers was performed according to well-established procedures.[4,5] The P(DEVP-stat-DAlVP-stat-VPA)-containing films were deposited on the static substrates by pipetting 50 µL of a 85 ppm (0.0085 wt.%) polymer-containing stock solution in methanol/water (6/1) onto the surfaces of either the QCM-D crystals or the silicon wafers. Immediately after adding the stock solution, the substrates were rotated at a spinning speed of 4000 rpm with an acceleration of 2500 rpm/s for 60 seconds.

Time-of-flight secondary ion mass spectrometry (ToF-SIMS)

Time-of-flight secondary ion mass spectrometry (ToF-SIMS) measurements were performed using an M6 instrument (Iontof, Münster, Germany) with a 30 keV Bi_3^+ primary ion beam operating at a target current of 0.6 pA. Spectra were calibrated using signals at known *m*/*z* values. Depth profiling was performed with a 2.5 keV Ar_{1300}^+ gas cluster ion beam (GCIB), applying a target current of 1 nA. Sputtering was carried out over a 500 by 500 µm area, while the analysis was conducted in the central 300 by 300 µm of the sputtered crater.

X-ray photoelectron spectroscopy (XPS)

X-ray photoelectron spectroscopy (XPS) analyses were conducted using a Supra+ system (Kratos, Manchester, UK) equipped with an Al K_α excitation source. The spectra were calibrated using the C 1s peak corresponding to C-C/C-H at 284.8 eV. The spin-coated QCM-D sensor sample was mounted on the sample holder using a silicone-free double-sided tape. Measurements were

performed at a 90° take-off angle, with a spot size of 300 by 700 μm and a pass energy of 20 eV for high-resolution spectra and 160 eV for survey spectrum. Data collection and analysis were performed using Kratos' ESCApe 1.5 software.

2. POLYMER AND HYDROGEL SYNTHESIS

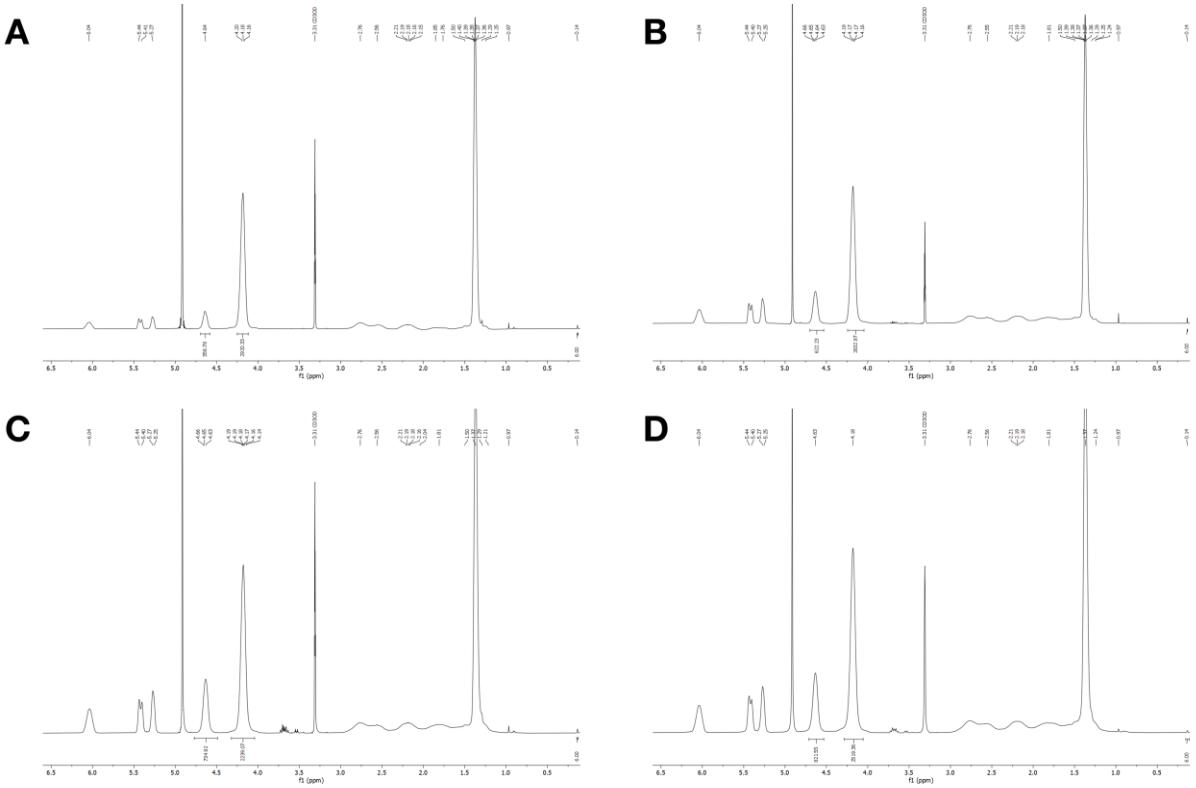

Figure S1: ^{1}H-NMR spectra of P(DEVP-stat-DAlVP) copolymers (Table 1, Entries 1-4) in CD_3OD with signals relevant for the molecular weight determination via ^{1}H-NMR spectroscopy (order A-D of the spectra corresponds to the order 1-4 in Table 1 of the manuscript).

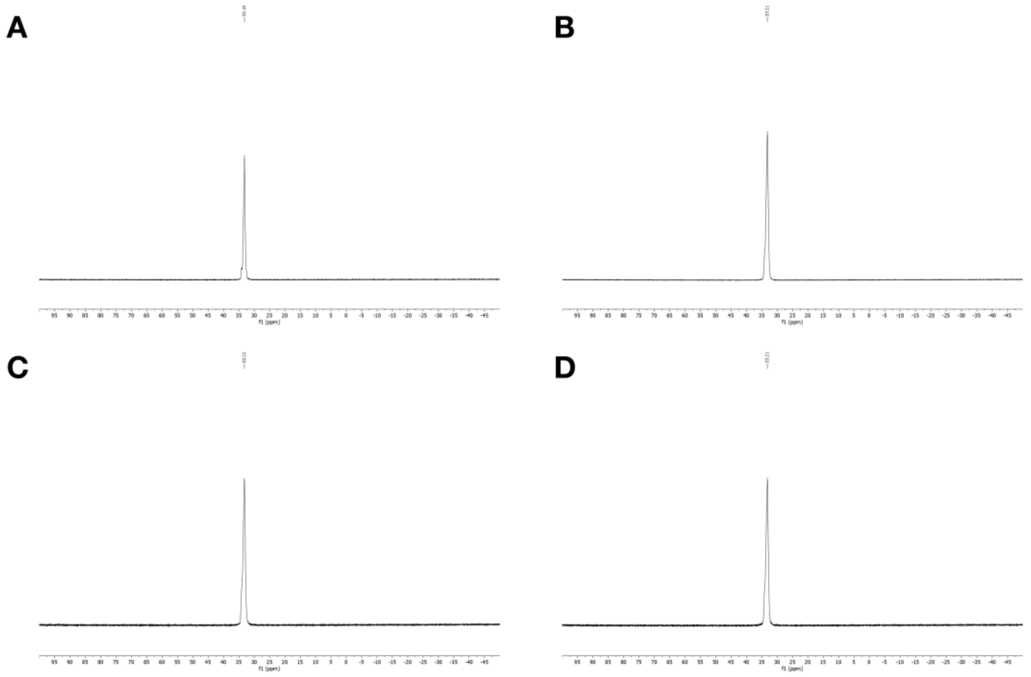

Figure S2: ^{31}P-NMR spectra of P(DEVP-stat-DAlVP) copolymers (Table 1, Entries 1-4) in CD_3OD (order A-D of the spectra corresponds to the order 1-4 in Table 1 of the manuscript).

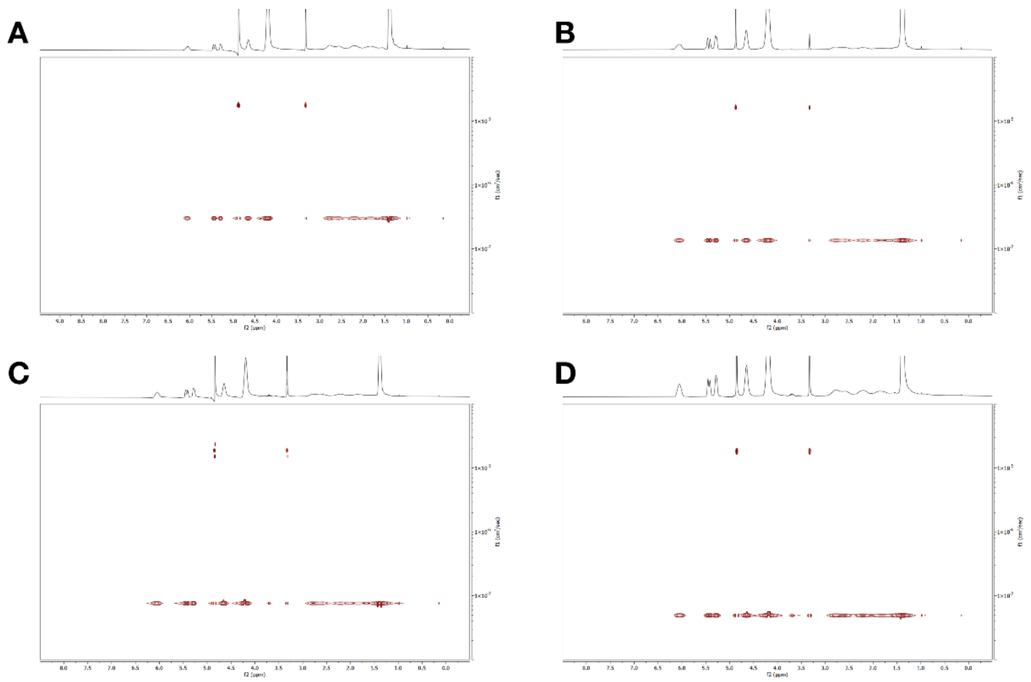

Figure S3: ^{1}H DOSY NMR spectra of P(DEVP-stat-DAlVP) copolymers (Table 1, Entries 1-4) in CD_3OD (order A-D of the spectra corresponds to the order 1-4 in Table 1 of the manuscript).

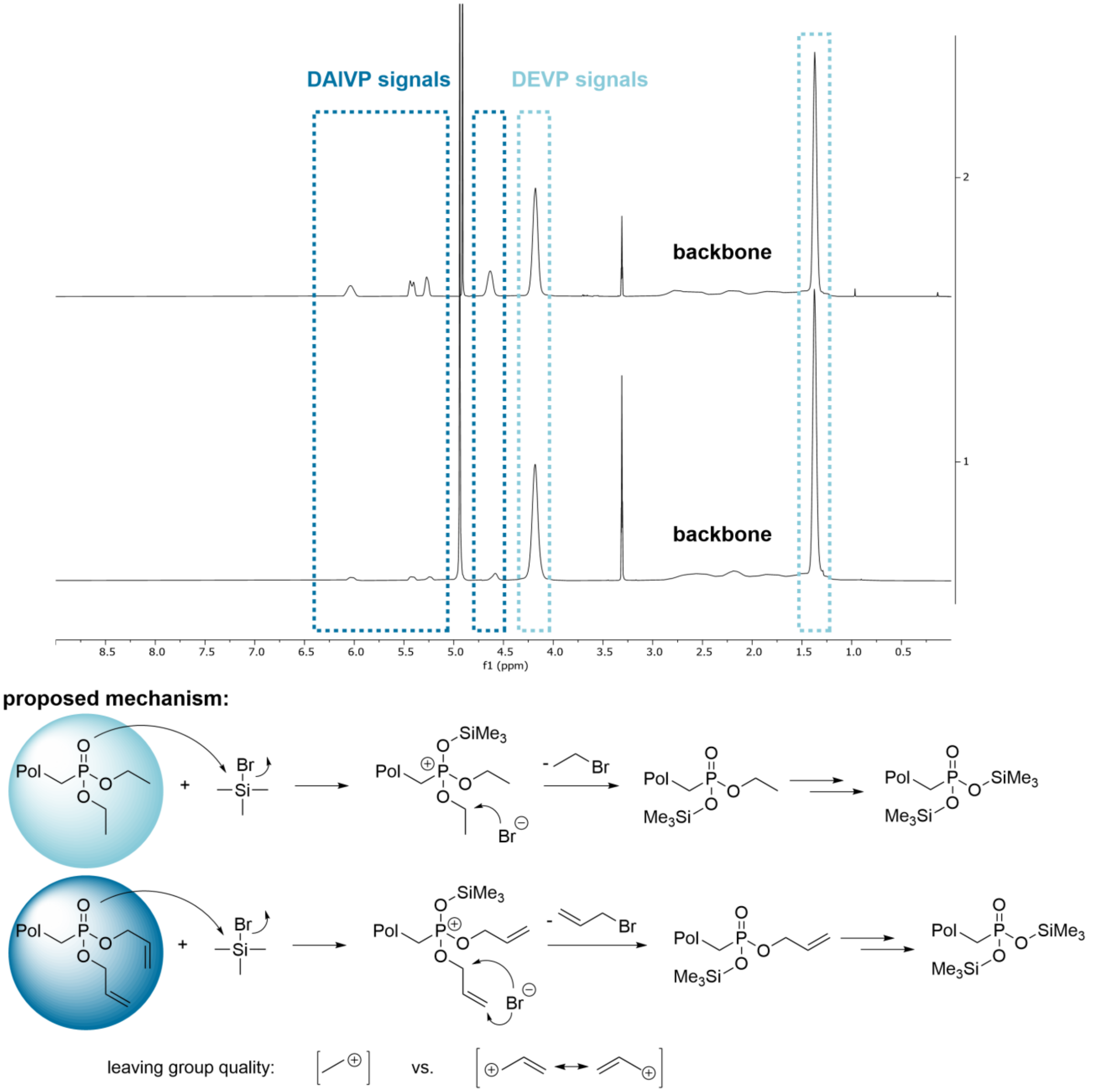

Figure S4: Comparison of ^{1}H-NMR spectra of P(DEVP-stat-DAlVP) (Table 1, polymer 2) (top spectrum) and P(DEVP-stat-DAlVP-stat-VPA) (Table 2, polymer 6) (bottom spectrum) in CD3OD (top) and proposed reaction mechanism for the polymer analogous transformation of copolymers by reaction with TMSBr (bottom).[6]

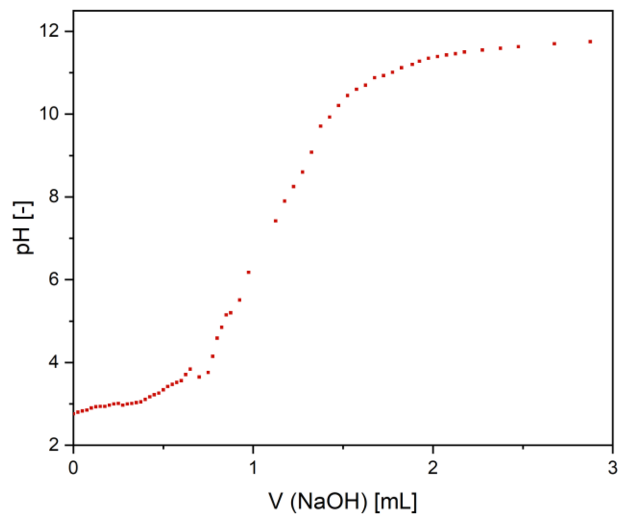

Figure S5: Titration of 10 mL of an aqueous solution poly(vinylphosphonic acid) (PVPA) (1 g L^{-1} polymer in H_2O, 9.3 mM VPA units) with 0.1 M NaOH (aq).

Hydrogel swelling experiments

For the determination of the water uptake, first, the dry weight of specimen subject to swelling experiments was determined after drying to weight constancy in vacuo. Subsequently, the samples were immersed in water for 8 hours. The swelling ratio given by Equation S1 was calculated by comparing the weight of the swollen specimen with the weight of the dry samples to obtain the water contents of each sample. Here, Q corresponds to the swelling ratio, M_s denotes the sample weight in the swollen state, and M_d refers to the weight of the specimen in the dry state. Further, standard deviations of the experiments were determined by performing the swelling experiments at least in triplicates.

$$Q = \frac{M_s - M_d}{M_d} \tag{S1}$$

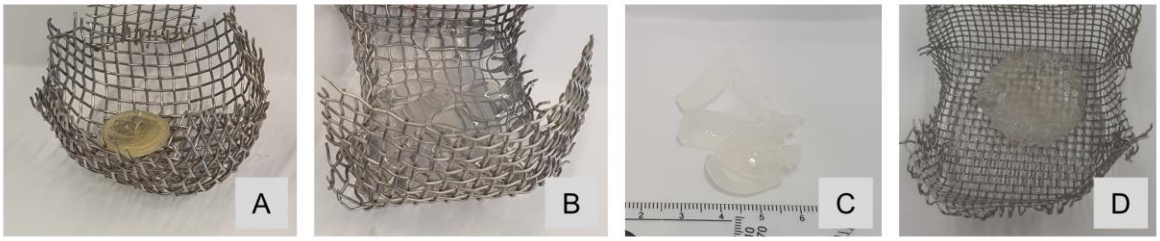

Figure S6: Images of hydrogel swelling (distilled water) of specimen obtained by photo-crosslinking of P(DEVP-stat-DAlVP-stat-VPA): hydrogel in the dry state (**A**), highly swollen hydrogel without structural integrity (Table 2, Entry 5) (**B**), swollen hydrogel forming a soft and brittle chip (Table 2, Entry 6) (**C**), and stable specimen after swelling (Table 2, Entry 8) (**D**).

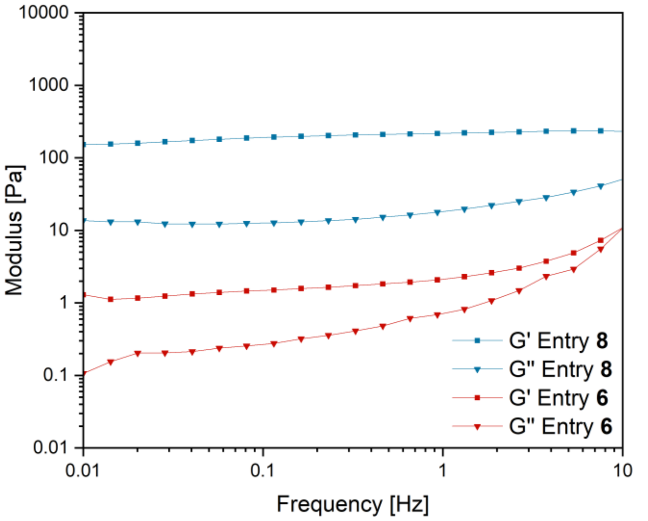

Figure S7: Comparison of the mechanical properties of hydrogels from polymers 6 (red curves) and 8 (blue curves) (Table 2) by frequency sweeps between 0.01 and 10 Hz at $\gamma = 1\%$.

3. THIN-FILM PREPARATION AND CHARACTERIZATION

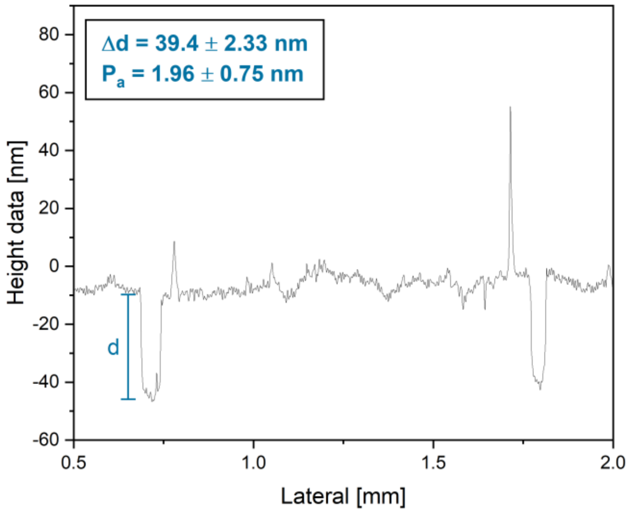

Figure S8: Exemplary profilometric measurement of a thin film of crosslinked P(DEVP-stat-DAlVP-stat-VPA) spin-coated on a silicon wafer and scratched twice with a thin razor blade.

Table S1: Summary of profilometric measurements to determine the dry film thickness and surface roughness of substrates spin-coated with crosslinked P(DEVP-stat-DAlVP-stat-VPA).

Substrate	**1**					
Δd [nm]	37.27	40.77	39.17	36.60	40.98	41.90
P_a [nm]	1.358		3.091		2.556	
Substrate	**2**			**3**		
Δd [nm]	37.577	38.234	35.136	41.522	41.841	42.386
P_a [nm]	1.110			1.671		

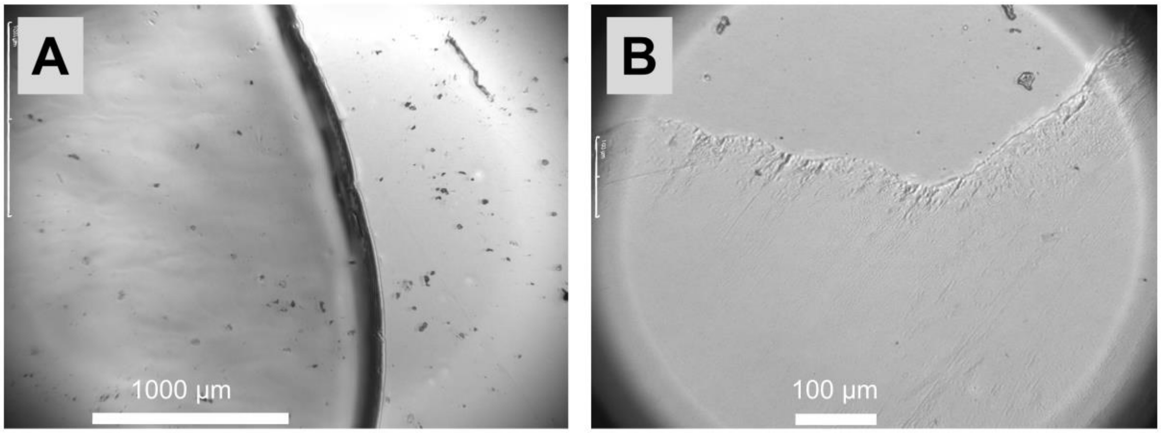

Figure S9: Light microscopic images of P(DEVP-stat-DAlVP-stat-VPA) spin-coated onto silicon wafers from an 85 ppm (0.0085 wt.%) solution in MeOH/H_2O (6/1) in two different magnifications: 5-fold magnification (**A**), and 20-fold magnification (**B**).

4. QUARTZ CRYSTAL MICROBALANCE MEASUREMENTS

Calculations of the mass deposition by the Sauerbrey equation

During the QCM-D measurements, the instrument simultaneously detects changes in the resonance frequency (Δf) and the energy dissipation (ΔD) caused by mass deposition or removal on an oscillating piezoelectric crystal. Dry film masses and masses of hydrogels coupled to the crystal during QCM-D experiments were calculated by applying the Sauerbrey equation, which is given by Equation S2:

$$\Delta m = -\frac{C \cdot \Delta f}{n} \tag{S2}$$

In the Sauerbrey equation, Δf is the measured frequency shift, C is the Sauerbrey constant (-17.7 ng Hz^{-1} cm^{-2} for the used 5 MHz crystals), n is the number of the overtone under consideration (n = 1, 3, 5, etc. considered automatically by the software) and Δm is the mass change of the crystal. The reported frequency changes Δf are normalized with respect to the third overtone number.

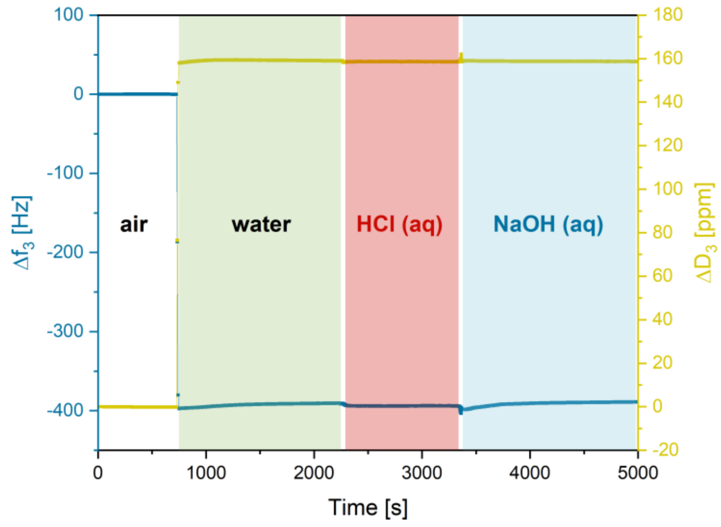

Figure S10: QCM-D measurement of empty crystals in different aqueous media and measurement on air before.

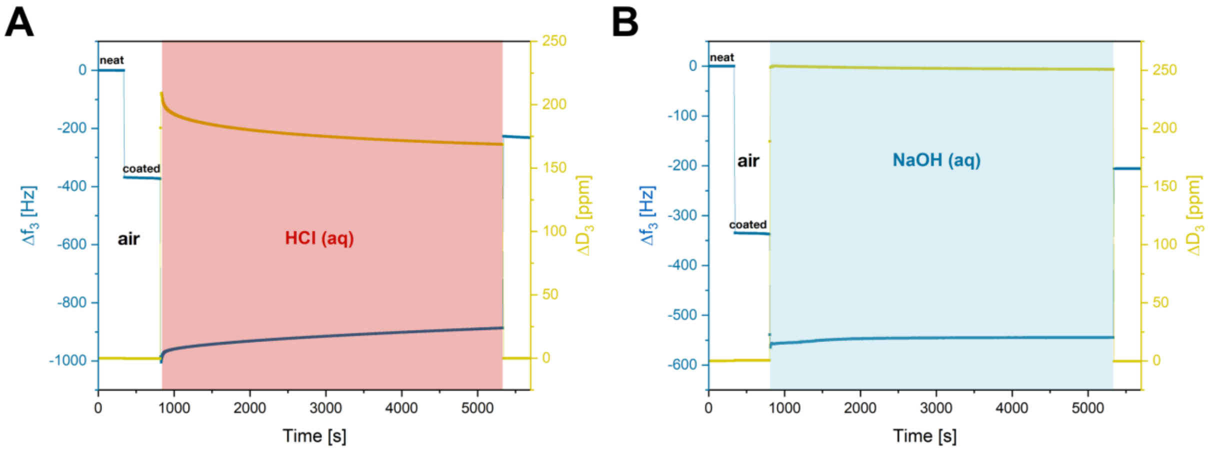

Figure S11: QCM-D measurements of thin films of non-crosslinked P(DEVP-stat-DAlVP-stat-VPA) in different aqueous media and measurements in air before and after exposure to the liquids, checking for potential leaching of samples. (**A**) Investigation of sample behavior under acidic conditions (pH 1) and (**B**) investigation of sample behavior under alkaline conditions (pH 13).

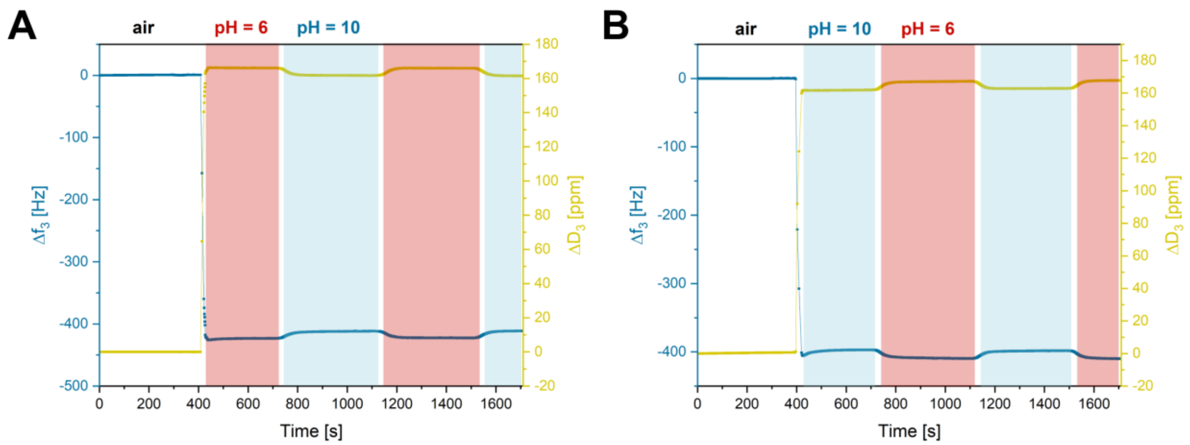

Figure S12: QCM-D measurement of uncoated sensors in air and different aqueous media. (**A**) Cycling of the pH value between pH 6 (0.1 M citrate buffer) and pH 10 (0.1 M carbonate buffer) over two cycles and (**B**) Cycling of the pH value between pH 10 (0.1 M carbonate buffer) and pH 6 (0.1 M citrate buffer) over two cycles.

5. REFERENCES

(1) Halama, K.; Schaffer, A.; Rieger, B. Allyl Group-Containing Polyvinylphosphonates as a Flexible Platform for the Selective Introduction of Functional Groups via Polymer-Analogous Transformations. *RSC Adv,* **2021**, *11* (61), 38555–38564. DOI: 10.1039/d1ra06452e. Published Online: Nov. 30, 2021.

(2) Salzinger, S.; Soller, B. S.; Plikhta, A.; Seemann, U. B.; Herdtweck, E.; Rieger, B. Mechanistic Studies on Initiation and Propagation of Rare Earth Metal-Mediated Group Transfer Polymerization of Vinylphosphonates. *J. Am. Chem. Soc.* **2013**, *135* (35), 13030–13040. DOI: 10.1021/ja404457f. Published Online: Aug. 21, 2013.

(3) Schaffer, A.; Kränzlein, M.; Rieger, B. Synthesis and Application of Functional Group-Bearing Pyridyl-Based Initiators in Rare Earth Metal-Mediated Group Transfer Polymerization. *Macromolecules* **2020**, *53* (11), 4345–4354. DOI: 10.1021/acs.macromol.0c00642.

(4) Mohan, T.; Kargl, R.; Doliška, A.; Vesel, A.; Köstler, S.; Ribitsch, V.; Stana-Kleinschek, K. Wettability and Surface Composition of Partly and Fully Regenerated Cellulose Thin Films From Trimethylsilyl Cellulose. *J. Colloid Interface Sci.* **2011**, *358* (2), 604–610. DOI: 10.1016/j.jcis.2011.03.022. Published Online: Mar. 12, 2011.

(5) Mohan, T.; Niegelhell, K.; Nagaraj, C.; Reishofer, D.; Spirk, S.; Olschewski, A.; Stana Kleinschek, K.; Kargl, R. Interaction of Tissue Engineering Substrates with Serum Proteins and Its Influence on Human Primary Endothelial Cells. *Biomacromolecules* **2017**, *18* (2), 413–421. DOI: 10.1021/acs.biomac.6b01504. Published Online: Jan. 24, 2017.

(6) Błażewska, K. M. McKenna Reaction–Which Oxygen Attacks Bromotrimethylsilane? *J. Org. Chem.* **2014**, *79* (1), 408–412. DOI: 10.1021/jo4021612. Published Online: Dec. 20, 2013.
